# Alternations in the foraging behaviour of a primary consumer drive patch transition dynamics in a temperate rocky reef ecosystem

**DOI:** 10.1111/ele.14064

**Published:** 2022-06-29

**Authors:** Joshua G. Smith, M. Tim Tinker

**Affiliations:** ^1^ Department of Ecology and Evolutionary Biology University of California Santa Cruz California USA; ^2^ National Center for Ecological Analysis and Synthesis University of California Santa Barbara California USA

**Keywords:** alternative stable states, behaviour, kelp forest, patch dynamics, trophic cascades

## Abstract

Understanding the role of animal behaviour in linking individuals to ecosystems is central to advancing knowledge surrounding community structure, stability and transition dynamics. Using 22 years of long‐term subtidal monitoring, we show that an abrupt outbreak of purple sea urchins (*Strongylocentrotus purpuratus*), which occurred in 2014 in southern Monterey Bay, California, USA, was primarily driven by a behavioural shift, not by a demographic response (i.e. survival or recruitment). We then tracked the foraging behaviour of sea urchins for 3 years following the 2014 outbreak and found that behaviour is strongly associated with patch state (forest or barren) transition dynamics. Finally, in 2019, we observed a remarkable recovery of kelp forests at a deep rocky reef. We show that this recovery was associated with sea urchin movement from the deep reef to shallow water. These results demonstrate how changes in grazer behaviour can facilitate patch dynamics and dramatically restructure communities and ecosystems.

## Graphical abstract

Understanding the role of animal behavior in linking individuals to ecosystems is central to advancing knowledge surrounding community structure, stability, and transition dynamics. Using 22‐years of long‐term subtidal monitoring, we show that an abrupt outbreak of purple sea urchins (Strongylocentrotus purpuratus) in southern Monterey Bay, California, USA, was primarily driven by a behavioral shift, not by a demographic response (i.e., survival or recruitment). We then tracked the behavior (passive or active grazing) of sea urchins for three years following the 2014 outbreak and found that behavior is strongly associated with patch state (forest or barren) transition dynamics. Image credit: Patrick Webster.

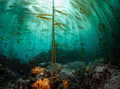

## INTRODUCTION

The importance of behaviour in linking individuals to ecosystems is widely recognised in the ecological literature (Ovadia & Schmitz, 2002; Schmitz, 1998; Sih et al., 2012; Werner & Peacor, 2003). Behaviour can facilitate community structure and functioning by altering the relative influence of key species interactions (e.g. competition, predation, mutualisms), changing the distribution of resources and through other non‐consumptive response pathways (Estes et al., 1998; Pace et al., 1999; Werner & Peacor, 2003). Although the debate continues over the relative importance of density versus behaviorally mediated influences of predators and primary consumers, both occur widely in nature and are often associated with trophic cascades (Beckerman et al., 1997; Kauffman et al., 2010; Schmitz et al., 1997; Werner & Peacor, 2003). Therefore, understanding how the presence of predators and resource availability reciprocally influence the behaviour of primary consumers is central to advancing knowledge of community structure, functioning, stability, and transition dynamics.

Sea urchin grazing in marine ecosystems around the world is often considered a fundamental driver of shifts from algal‐dominated habitats to alternative sea urchin ‘barrens’ that are void of macroalgae (Filbee‐Dexter & Scheibling, 2014; Ling et al., 2015). These shifts have profound consequences on the structure and functioning of coral reefs, seagrass, kelp forest and rocky intertidal ecosystems (Done, 1992; Filbee‐Dexter & Scheibling, 2014; Watson & Estes, 2011). Resource availability and predator‐driven impacts are perhaps the two most well‐documented factors known to influence patterns in sea urchin grazing behaviour (Burt et al., 2018; Cowen, 1983; Harrold & Reed, 1985; Mann, 1982). Cascading effects resulting from the loss of sea urchin predators provide strong evidence of density‐mediated indirect interactions (Burt et al., 2018; Estes et al., 1998), whereas reductions in the availability of food or risk‐cues have been associated with behaviorally mediated indirect interactions (Harding & Scheibling, 2015; Spyksma et al., 2017). However, the relative influence of these factors is often context‐dependent and difficult to decouple from other more environmentally driven processes such as how grazers respond to substrate complexity, seasonality, swell and water temperature (Konar et al., 2014; Randell et al., 2022; Vivian‐Smith, 1997). Therefore, the factors that contribute to modifications in sea urchin grazing behaviour can have important implications for the state of communities and ecosystems.

In temperate kelp forest ecosystems, sea urchin behaviour can be categorised into two fundamental modalities. In kelp forests, where abundant detrital (i.e. ‘drift’) algae are deposited in crevices, urchins mainly employ a cryptic passive‐grazing modality (Duggins et al., 1989; Krumhansl & Scheibling, 2012; Sala & Zabala, 1996). The presence of predators may also elicit a direct response in sea urchins that influence cryptic behaviour or indirectly by maintaining forests (and therefore abundant drift) through trophic cascades (Cowen, 1983; Estes et al., 1998). However, when the production of detrital kelp is limited, sea urchins fundamentally shift their behaviour to an active grazing modality, where they emerge from the refuge and roam on an open reef surface in search of live macroalgae (Harrold & Reed, 1985; Kriegisch et al., 2019). Additionally, because sea urchins have a dispersive larval‐stage life history, kelp‐urchin dynamics can also be strongly driven by spatially explicit and episodic recruitment (Lafferty & Kushner, 2000; Okamoto et al., 2020).

Kelp forests along the west coast of North America recently experienced a rapid and pronounced shift from highly expansive forests to unproductive sea urchin barrens. Starting in late 2013, a coast‐wide sea star epizootic decimated the urchin predator *Pycnopodia helianthoides* (hereafter, *Pycnopodia*), followed by an episodic marine heatwave event that occurred from mid‐2014 into 2016 (Harvell et al., 2019; McPherson et al., 2021). Shortly after (2014–2016), large‐scale reductions in kelp biomass were recorded along the mainland coasts of California, the United States and Baja California, Mexico (Beas‐Luna et al., 2020), with pronounced urchin outbreaks occurring in central and northern California (McPherson et al., 2021; Smith et al., 2021). In northern California where bull kelp (*Nereocystis luetkeana*) is the dominant structure‐forming foundation species, over a 95% reduction in historical kelp biomass was documented (McPherson et al., 2021; Rogers‐Bennett & Catton, 2019). Similar large‐scale loss of kelp biomass was recorded at the southern range limit of the giant kelp (*Macrocystis pyrifera*) near Bahía Asunción, Mexico (27.1°N; Arafeh‐Dalmau et al., 2019, Beas‐Luna et al., 2020). However, along the central coast of California, giant kelp‐dominated forests experienced a shift to a patchy mosaic of remnant forests interspersed with sea urchin barrens (Smith et al., 2021). As such, whether the observed 2014 sea urchin outbreak resulted from a behavioural shift (i.e. from passive grazing of detrital algae to active grazing on live macroalgae) or from changes in recruitment remains unresolved.

In this study, we explore whether an outbreak of purple sea urchins that occurred in 2014 along the Monterey Peninsula, CA, USA was driven by a behavioural shift (i.e. emergence from refuge or redistribution following the regional extirpation of *Pycnopodia* and reduced productivity of kelp associated with the marine heatwave) or by a demographic response (i.e. changes in survival or recruitment). We then tracked the behaviour of sea urchins in the years following the 2014 outbreak to determine how grazer behaviour shapes alternations between kelp‐dominated (hereafter, ‘forested’) and urchin‐dominated (hereafter, ‘barren’) states. This study was motivated by the following hypotheses: (1) sea urchins emerged from refuge following the regional collapse of *Pycnopodia*, the 2014–2016 marine heatwave, and a decline in kelp production, (2) sea urchin behaviour (passive or active) explains patch state (forested or barren) transition dynamics and (3) sea urchin migration in search of alternative food sources is associated with macroalgae recovery.

## MATERIALS AND METHODS

### Study system

**FIGURE 1:**
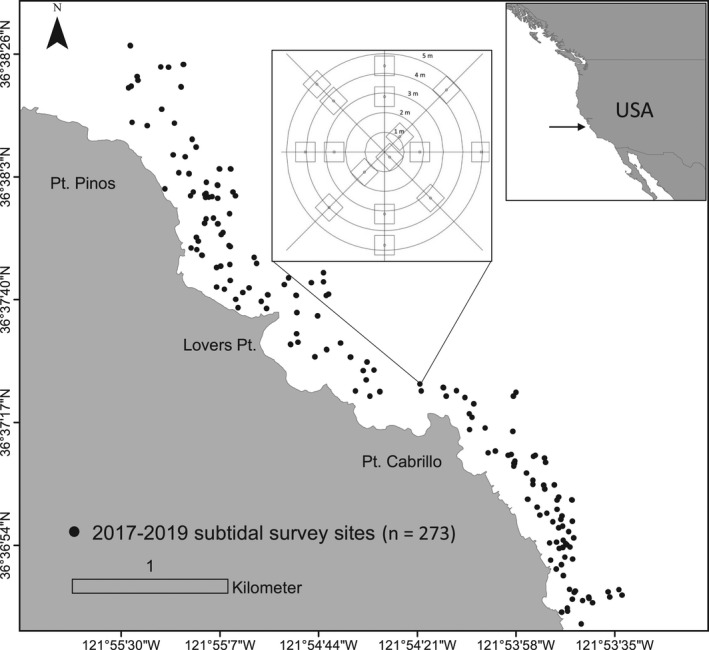
Study area along the Monterey Peninsula, California, USA. Each point represents an independent replicate survey site sampled from 2017 to 2019. The inset diagram depicts the sampling design used to survey each site using eight 5‐m long transects (lines) radiating from a fixed central position, with two 1 m^2^ quadrats (squares) sampled per transect

This study was conducted in the nearshore temperate reefs of southern Monterey Bay, California, USA (Figure 1). All marine algae and invertebrates within the survey region are protected from harvest within marine protected areas. The giant kelp, *Macrocystis pyrifera*, is the dominant habitat‐forming algae and the purple sea urchin, *Strongylocentrotus purpuratus* (hereafter, ‘sea urchin’), is the principal benthic herbivore, although bull kelp (*Nereocystis luetkeana*) and red sea urchins (*Mesocentrotus franciscanus*) also inhabit the region. In 2013, a coastwide sea star wasting syndrome occurred that extirpated the urchin predator *Pycnopodia helianthoides* throughout California by 2014 (Harvell et al., 2019). Shortly, thereafter, a dramatic increase in visually detectible sea urchins shifted the region into a patchy mosaic of remnant forests interspersed with sea urchin barrens (Smith et al., 2021). We used long‐term benthic subtidal monitoring data to determine whether the initial sea urchin outbreak was primarily evidenced by a behavioural shift or by a demographic response (i.e. sea urchin recruitment or survivorship). We then conducted a series of separate surveys over the course of 3 years to explore whether (and how) sea urchin behaviour is associated with patch state transition dynamics across the mosaic.

### Sea urchin behavioural shifts at the regional scale

To determine the temporal point when the sea urchin outbreak occurred, we conducted change‐point analyses on a 22‐year time series of published subtidal data collected by the Partnership for Interdisciplinary Studies of Coastal Oceans (PISCO; Malone et al., 2021). Briefly, we used all 12 PISCO sites (Figure S1) surveyed annually between mid‐June to mid‐October from 1999 to 2021 in southern Monterey Bay, California. Annual surveys at each site consisted of visual surveys by SCUBA divers of the density and percent cover of conspicuous benthic algae, invertebrates, and benthic and water column‐dwelling fishes. Density and percent cover estimates of conspicuous benthic algae and invertebrates were recorded along with six replicate 2 × 30 m transects stratified across three bottom depths (5, 12.5, 20 m; two transects per depth level). We used a segmented regression with multiple change points to determine the most likely temporal change point locations on the mean transect density (no. per 60 m^2^) of sea urchins surveyed in sequential years.

To determine whether the sea urchin outbreak is explained by a shift in sea urchin behaviour from passive to active grazing, we examined the annual size frequency (test diameter recorded to the nearest 1 cm) of sea urchins recorded on PISCO surveys from 2011 to 2016 (sea urchin sizing began in 2011). Support for the behavioural shift hypothesis would be evidenced by increases in counts of all size classes of urchins as they emerged from crevices and became more visible on surveys. Alternatively, support for the recruitment hypothesis (i.e. that the urchin outbreak was associated with a recruitment event) would be evidenced by a dramatic and disproportionate increase in the frequency of sea urchins less than 3 cm. We used a Kolmogorov–Smirnov test to evaluate equality in urchin size distribution across the critical 2013–2014 breakpoint (identified from the segmented regression). Finally, we tested for a relationship between urchin counts and terrain ruggedness (i.e. reef complexity), reef slope and relief among which we found no effect (Supplementary Information Methods).

### Population state dynamics

To further explore whether the marked increase in counts of adult (>3 cm) sea urchins after 2013 is explicable by a sea urchin behavioural shift, we used a Bayesian hierarchical framework to fit a size‐structured population dynamics model to survey data. We used this model to evaluate sea urchin recruitment, survival (growth) and detection probability as plausible drivers of observed population dynamics. The structure of the process model is described in detail in Supplementary Information Methods. Briefly, the key demographic processes were the annual rates of growth, survival and recruitment, while a fourth observer process (detection probability) related the latent dynamics of the true population to the observed survey data. We assume that detection probability is reflective of sea urchin behaviour (i.e. whether sea urchins are cryptic and not counted on surveys or actively grazing and visible to divers). The model tracked the dynamics of 10 size classes (corresponding to 1 cm size increments), with growth transition probabilities between size classes parameterised from literature‐reported values (Ebert, 2010). Model‐estimated parameters included baseline values of recruitment, survival and size‐specific detection probability as well as year‐to‐year differences in each of these processes over the study period (1999–2021) that were estimated as hierarchical random effects.

We used standard Markov Chain Monte Carlo methods (MCMC) to fit the process model to survey data on average size‐structured urchin counts per transect (details of fitting methods and diagnostics are described in detail in Supplementary Methods). We summarised the estimated proportional changes in recruitment, survival and detection probability after 2013, and we used a simulation‐based sensitivity analysis to calculate the relative contribution of variation in each of these processes to the observed increase in urchin counts over the study period.

### Patch‐level dynamics

We explored patch‐level dynamics over a 3‐year study period (2017–2019) using additional field surveys to further examine how sea urchin behaviour underpins switching among patch states (forest, barren). Survey sites were initially randomly selected and sampled annually from May to September in 2017 (*n =* 90), 2018 (*n* = 111) and 2019 (*n* = 72) to determine patch transition dynamics and attributes of each patch state such as sea urchin behaviour, density and the cover of key groups of algae (Figure 1). Survey sites were randomly selected on hard substratum between 5 and 20 m of water (based on diving limitations) and consistent efforts were made to replicate spatial sampling through time (i.e. survey sites were resampled in 2018 and 2019). A site consisted of 16 1 m^2^ quadrats randomly stratified across eight 5‐m long transects (two quadrats per transect), and each transect radiated from a fixed central location (Figure 1). Therefore, each survey site represents an independent replicate sample.

The state (barrens, forest) of each site was characterised by constructing a linear discriminant analysis (LDA) using urchin behaviour (density exposed and concealed), density and the percent cover of algae as classifiers. In the field, each site was surveyed using 16 randomly placed 1 m^2^ quadrats fixed with a high‐resolution GoPro Hero4 camera and two Sola LED video lights. The density of urchins was recorded in situ within each quadrat by quantifying visually detectible sea urchins and by searching in cracks and crevices for cryptic individuals. We also recorded site patch states (barrens, forest) based on initial impressions of the site at the start of each dive. In the lab, photoquadrats were analysed to determine the number of actively foraging (i.e. exposed) sea urchins and to estimate the cover of key algal groups that are characteristic of forests and barrens. Each photoquadrat was assigned 16 universal points using a digital grid in ImageJ. Because many algae are difficult to visually quantify to the species level in imagery, we used four taxonomic categories that are known indicators of patch state (Filbee‐Dexter & Scheibling, 2014): articulated coralline algae, encrusting coralline and red algae, brown algae and foliose red algae. Finally, exposed sea urchins of all detectible sizes were quantified from photoquadrats by counting only urchins where 50% or more of the test diameter was visible (Smith et al., 2021). We then constructed the LDA by using these variables (sea urchin density, proportion of exposed urchins, cover of algae) as predictors of the field patch‐state classification. Out of 284 sites used in training the LDA, only seven were misclassified and the entropy *R* ^2^ was 0.89. Therefore, we elected to use the predicted states from the LDA (rather than diver‐based site impressions) in subsequent analyses.

To test the hypothesis that shifts in patch state are associated with alternations in sea urchin foraging behaviour, we explored transition dynamics across two time steps (2017–2018 and 2018–2019). For this analysis, only sites surveyed at the same spatial location in sequential years were used to determine whether each site (1) persisted in the same state across the time step, (2) forward‐shifted from a forest to barren or (3) reverse‐shifted from a barren to a forest. Logistic regression was used to determine the transition probability based on the natural log‐transformed mean density of exposed (i.e. actively foraging) sea urchins, the mean number of cryptic urchins and starting state (barren, forest). We defined the logistic target level based on a positive state shift, where a transition to a different state in the following year was classified as ‘1’ and state persistence as ‘0’. Therefore, each of the variables in the model represents starting‐year values used to predict the following‐year state. We used AIC model selection to identify the best‐fit relationship between patch transition and the density of sea urchins exposed or concealed. Finally, to determine the strength of discontinuity in state‐shift thresholds, we examined the logged odds of state transition probabilities as a function of exposed sea urchin density.

### Forest recovery following sea urchin movement

In 2019, we observed a dramatic reduction in counts of sea urchins and an extraordinary recolonisation of a kelp forest to an area that was an expansive sea urchin barren just 2 years prior (2017). Surveys farther inshore (i.e. shallow water) during the recovery year (2019) revealed an abundance of large (>6 cm) sea urchins and a reef devoid of macroalgae. We hypothesised that the observed recovery of kelp to the deep reef area was associated with sea urchin movement to shallow water because the shallow reef was previously dominated by red foliose algae, an alternative sea urchin food source. To test this hypothesis, we examined urchin size structure and density across three depth zones and three survey years. We categorically assigned all the survey sites (*n =* 18) near the recovery area to one of three depth zones: shallow (0–6 m), mid (7–13 m) and deep (14–20 m). Sea urchins were categorically assigned to one of three size classes based on test diameter: small (<30 mm), medium (30–38 mm) and large (>38 mm). These size classes were selected based on the first, second and third quantiles of the entire population size distribution across all three survey years (*n =* 6827 individuals).

We used a mixed model with a Restricted Maximum Likelihood (REML) to test for differences in mean sea urchin density across three survey years (2017–2019), three depth zones (shallow, mid and deep) and three size classes of sea urchins (small, medium and large). The model was constructed as a full factorial with year, depth zone and size class as fixed effects and site and transect as random effects. We then used a contrast test to examine the hypothesis that the density of large‐ and medium‐sized urchins declined over the period 2018–2019 in the deep zone and simultaneously increased in the shallow zone. The output of the mixed model revealed that the density of sea urchins did not significantly change across the 2017–2018 time period. Therefore, we restricted the subsequent contrast test to the 2018–2019 period.

Finally, we explored changes in the algal assemblage across each depth zone in relation to sea urchin movement. To test for changes in the mean percent cover of foliose red algae, brown algae and encrusting algae, we used an analysis of variance (ANOVA) test on photoquadrats at each survey site with depth zone and algae type as predictors of percent cover. We then used PISCO data on kelp density from an adjacent site located near the recovery area (Pt. Pinos) to examine whether the spatial and temporal kelp recovery at deep reefs was associated with sea urchin movement to shallow water.

## RESULTS

### Sea urchin behavioural shifts at the regional scale

**FIGURE 2:**
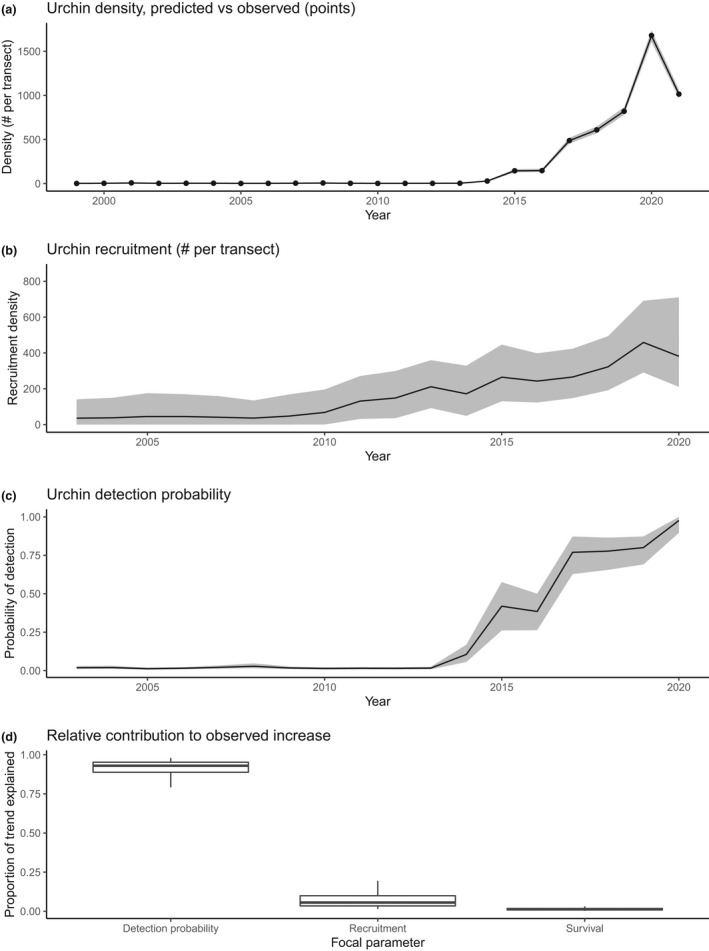
Trends in purple sea urchins at long‐term PISCO subtidal monitoring sites in Monterey Bay, California, USA (Malone et al., 2021). (a) Projections of mean urchin density trends (number per 60 m^2^ transect) between 1999 and 2021 based on a Bayesian population model, showing mean estimates (solid line), 90% credible interval (grey shaded band) and raw data (solid points). (b) Trends in urchin recruitment density based on the results of a Bayesian population model (solid line shows mean estimates and shaded band shows 90% credible interval). (c) Trends in detection probability for large urchins based on the results of a Bayesian population model (solid line shows mean estimates and shaded band shows 90% credible interval). (d) Boxplot of results from a simulation‐based sensitivity analysis, showing the relative contribution of three processes to the observed increase in observed urchin density after 2014: 1) detection probability, 2) annual recruitment rate and 3) annual survival rate

**FIGURE 3:**
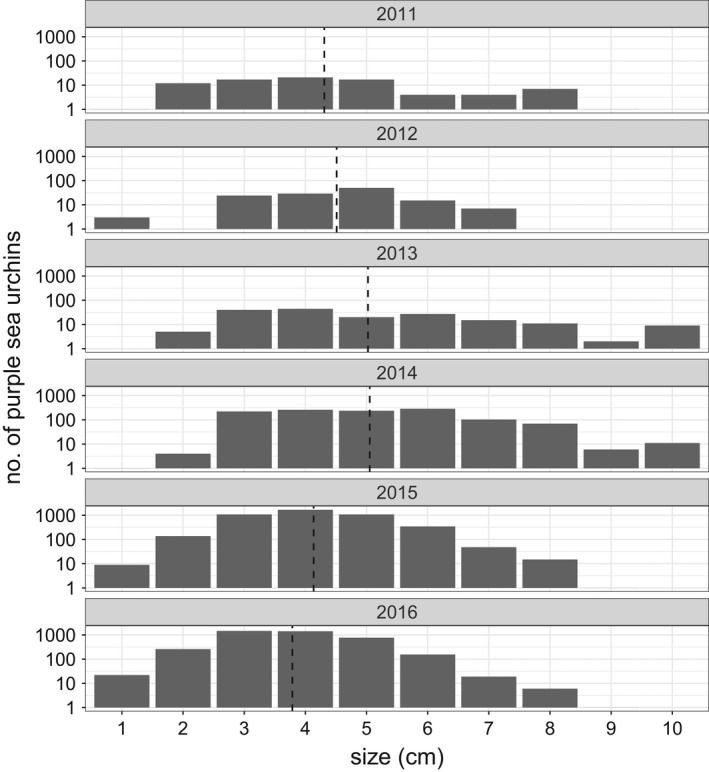
Size frequency distribution of purple sea urchins (*Strongylocentrotus purpuratus*) in Monterey, CA recorded on long‐term subtidal monitoring surveys by PISCO (Malone et al., 2021). Each bar depicts the log‐number of measured purple sea urchins recorded across 12 discrete size classes (test diameter). Vertical dashed lines indicate the mean size for a given year, weighted by counts for each size class

A sharp increase in the density of visually detectible purple sea urchins was initiated in 2014 and continued for at least 6 years before reaching an apparent deceleration (Figure 2a). Prior to 2014, the mean density of visually detectible sea urchins was 3.18 individuals (per 60 m^2^ transect). However, the density of visually detectible sea urchins in the 2015–2020 period markedly increased to over 700 individuals (per 60 m^2^ transect). Despite the dramatic increase in the density of visually detectible sea urchins, the size frequency distribution of urchins between 2013 and 2014 was similar, although larger urchins (>4 cm) were more frequently detected in 2014 (Figure 3). Moreover, we did not find evidence of an anomalous pulse in urchins at the lower end of the size distribution (<4 cm) in the years prior to 2014.

The size‐structured population model converged well (r‐hat <1.05 for all fitted parameters) and provided excellent goodness of fit to the survey data, satisfying all posterior predictive checks (Figure S4 and Table T3). The resulting hindcast estimates of density trends and annual size‐frequency distributions closely matched field survey estimates (Figure 2a, Figure S7). The model results indicated an increasing rate of recruitment beginning after 2010 (Figure 2b) and an even more dramatic increase in detection probability beginning after 2013 (Figure 2c). The mean detection probability for large urchins increased 37‐fold after 2013, as compared to a 5‐fold increase in recruitment and no significant change in survival rates (Figure S8). Variation in detection probability alone was able to explain 93% of the observed increase in urchin counts compared to approximately 6% explained by variation in recruitment and 1% explained by variation in survival (Figure 2d). These results suggest that the dramatic increase in counts of purple sea urchins that began in 2014 was explained mostly by a change in the probability that urchins of all size classes were detected, consistent with a behavioural shift (e.g. the emergence of urchins from the refuge).

### Patch‐level dynamics

We found support for the hypothesis that patch state transitions are explained by shifts in sea urchin behaviour. The linear discriminant analysis revealed that 113 sites persisted as the same starting and ending state across time steps, 11 forward‐shifted from a forest state to barren and 12 reverse‐shifted from a barren state to the forest (Figure S9). Model selection for the full logistic regression with behaviour (active, passive) as a predictor of the year‐following state showed that the density of actively foraging (i.e. exposed) urchins was the strongest relative determinant of transition probability (*R* ^2^ = 0.18, *p* < 0.0001, AICc = 108, 𝚫AIC = 10).

**FIGURE 4:**
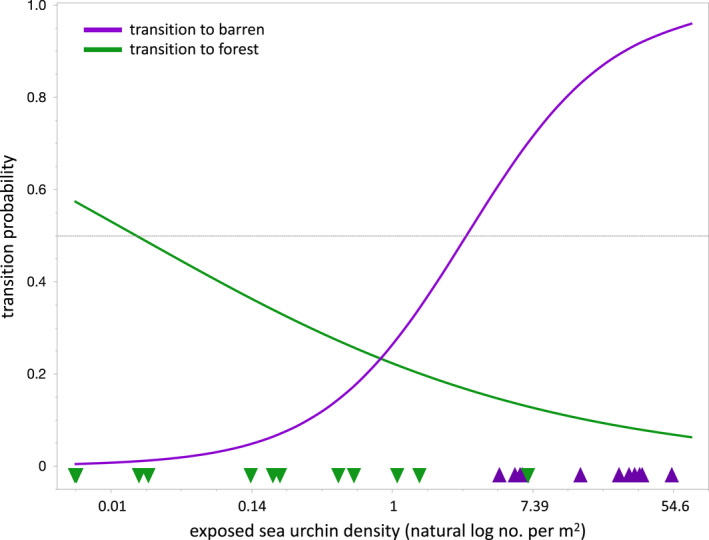
Transition probabilities depicting simulated logged odds for each starting state based on the log‐transformed density of exposed sea urchins. The purple line depicts the probability of a forest patch transitioning to a sea urchin barren in the following year based on the starting density of exposed sea urchins, and the green line depicts the probability of a barren becoming a forest in the following year. Also included are triangles that depict the final density (no. per m^2^) of exposed sea urchins for patches that transitioned (green triangles are patches that became forests, purple are patches that became barren). The dashed horizontal line indicates the 50% probability transition threshold

An analysis of the logged odds from the logistic regression revealed evidence of a strong discontinuous state shift (Figure 4). The 50% probability transition threshold for a forward shift from a kelp forest to a sea urchin barren was 2.71 exposed urchins/m^2^. However, the 50% probability transition threshold for a reverse shift from a barren to a forest was 0.03 exposed urchins/m^2^.

### Forest recovery following sea urchin movement

**FIGURE 5:**
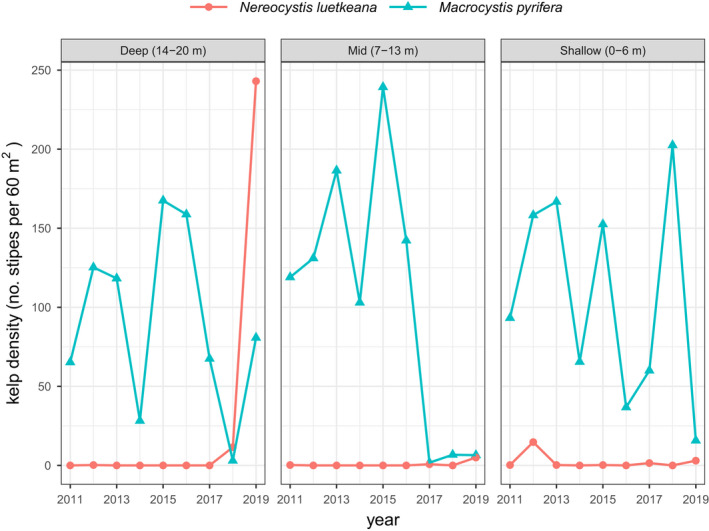
Kelp dynamics at Pt. Pinos, California, USA. Each line depicts the annual mean density (per 60 m^2^ transect) of giant kelp (*Macrocystis pyrifera*, blue) and bull kelp (*Nereocystis luetkeana*, orange) across three depth zones recorded by PISCO (Malone et al., 2021)

In 2019, we observed a remarkable recovery of forests at a deep (14–20 m) rocky reef that was an expansive sea urchin barren just 2 years prior (2017). Long‐term subtidal monitoring data revealed a rapid decline in kelp density across all depth zones in 2014 that coincided with the onset of sea urchin barrens. Starting in 2018, bull kelp (*Nereocystis luetkeana*) recolonised the deep depth zone, with a sharp uptick in 2019 (Figure 5; Figures S10 and S11).

**FIGURE 6:**
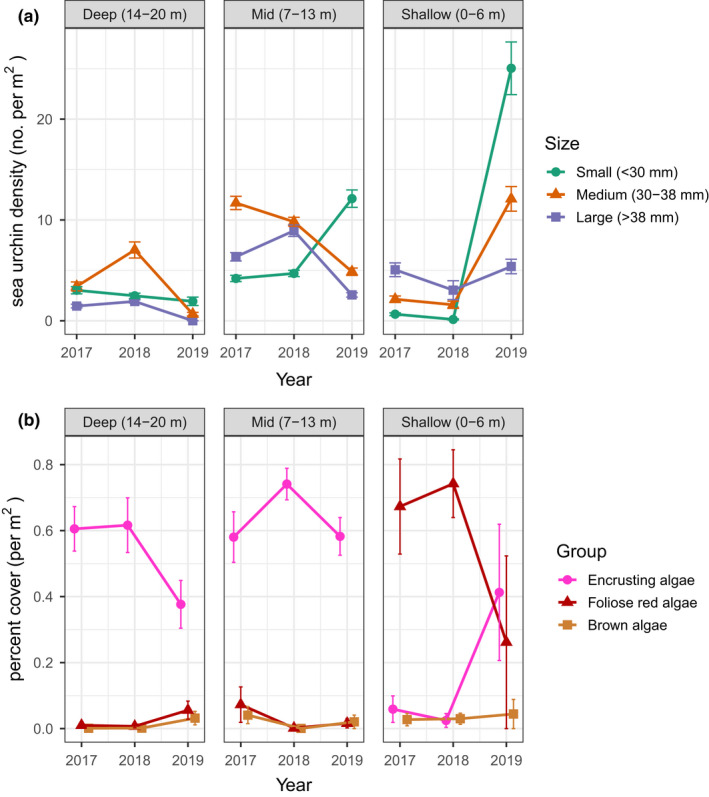
Urchin movement dynamics and algae per cent cover across three depth zones. Panel (a) depicts the mean density of small (<30 mm), medium (30–38 mm), and large (>38 mm) purple sea urchins. Panel (b) depicts the mean cover of foliose red algae (red), encrusting red and coralline algae (pink) and brown algae (brown). Error bars are included as 95% confidence intervals

We found that the total density of sea urchins significantly decreased in the deep zone between the 2018 and 2019 sampling seasons, with the most pronounced effects occurring in the large and medium urchin size classes (*R* ^2^ = 0.53, *p* < 0.0001; Figure 6a). A less dramatic but similar decline in medium and large urchins was observed across the same time step at mid‐depths (7–13 m). In the shallow zone, the total mean sea urchin density increased from 1.58 urchins/m^2^ (±0.34 SE) to 14.17 urchins/m^2^ (±1.19 SE). All size classes of urchins significantly increased in the shallow zone in the 2019 survey year (*p* < 0.0001). The contrast test revealed that the density of large‐ and medium‐sized urchins significantly declined within the deep zone between 2018 and 2019 (*t* ratio = 2.77, *p* < 0.005) and increased within the shallow zone during this same time period (*t* ratio = −5.69, *p* < 0.0001). Finally, a comparison of slopes between the deep and shallow zones across the 2018 and 2019 periods revealed that they were significantly different (DenDF = 2765, *F* = 20.16, *p* < 0.0001).

Results from analyses on the percent cover of foliose red algae, brown algae and encrusting algae further support the hypothesis of mass sea urchin movement to shallow water. Starting in 2019, the cover of encrusting coralline algae significantly decreased in the deep zone, along with a simultaneous increase in the cover of foliose red and brown algae (*R* ^2^ = 0.79, DF = 2, *p* < 0.0001; Figure 6b). In the shallow zone, the cover of foliose red algae significantly decreased, with a pronounced uptick in the cover of encrusting coralline algae (*R* ^2^ = 0.33, DF = 2, *p* < 0.0001).

## DISCUSSION

This study demonstrates the important role of grazer behaviour in facilitating patch‐state transition dynamics. The kelp forest‐urchin barrens mosaic that developed following the extirpation of *Pycnopodia* and the marine heatwave revealed how grazer behaviour shapes alternations between kelp‐dominated and urchin‐dominated states. These findings suggest that the initial 2014 sea urchin outbreak along southern Monterey Bay, California was primarily driven by the emergence of adult sea urchins from refuge, not by a demographic response (i.e. recruitment). Behaviorally driven alternations among patch states across the mosaic further demonstrate the role of grazer behaviour in facilitating transition dynamics.

In many systems, behaviour is a primary mechanism for the organisation of ecological communities (Karatayev et al., 2021; Lima & Zollner, 1996; Werner & Peacor, 2003). However, behavioural‐driven community patterning often results from demographic (i.e. recruitment) or density‐dependent responses of predators and their prey (Levin, 1976). Our study supports how multiple biotic (e.g. recruitment, loss of key predators) and environmental (e.g. marine heatwaves, grazer metabolic responses to warming events) perturbations may interact to influence behavioural switching that can facilitate persistent patterning of community states. The initial sea urchin outbreak observed in this region in 2014 is likely reflective of a shift in grazing modality (from passive to active grazing), potentially in response to several coinciding factors such as reduced food availability, increased metabolic demands from the warming event (e.g. Rasher et al. 2020), recruitment leading to adult behavioural switching and from a reduction in the abundance of a benthic mesopredator (Burt et al., 2018; Cowen, 1983; Harrold & Reed, 1985).

While we did not find strong evidence of a demographic response coinciding with the 2014 sea urchin outbreak, recruitment facilitation is a known driver of alternative state dynamics (Baskett & Salomon, 2010; Karatayev et al., 2020). Sea urchin recruitment dynamics are often episodic, with considerable geographic variation (Ebert & Russell, 1988; Okamoto et al., 2020; Pearse & Hines, 1987). Following the initial sea urchin behavioural shift in 2014, it is possible that the formation of barren patches enhanced sea urchin recruitment to barrens within the mosaic. Another alternative hypothesis is that increased sea urchin recruitment may have led to the observed behavioural response in adults, especially after 2014. Additionally, recruitment may have occurred prior to 2012 in this system or with variable timing and magnitude at other locations along the northeastern Pacific Ocean (Okamoto et al., 2020; Rogers‐Bennett & Catton, 2019).

Long‐term monitoring observations along the central coast of California, USA indicated that the 2014 sea urchin outbreak continued for at least 6 years and was potentially reinforced by recruitment after 2014. During this same period, canopy‐forming kelps to the north and south of the study region experienced unprecedented declines resulting from the marine heatwave and even more expansive outbreaks of purple sea urchins (Arafeh‐Dalmau et al., 2019; Beas‐Luna et al., 2020; McPherson et al., 2021; Rogers‐Bennett & Catton, 2019). One explanation for the persistence of remnant kelp patches in this system (as opposed to adjacent neighbouring areas) is the presence of trophically redundant predators. The urchin predator guild along the west coast of North America is comprised of six key species: sea otters (*Enhydra lutris nereis*), lobsters (*Panulirus interruptus*), sheephead (*Semicossyphus pulcher*), sunflower sea stars (*Pycnopodia helianthoides*), rock crab (*Cancer* spp.) and wolf eels (*Anarrhichthys ocellatus*; Scheibling & Hamm, 1991, Eisaguirre et al., 2020). The abundance of these species varies geographically along the west coast. In northern California, where rock crab and wolf eels are the only alternative predators of urchins, forests were reduced by over 95% with the loss of *Pycnopodia* (McPherson et al., 2021). However, forests in southern California that have a suite of urchin predators (e.g. lobster, sheephead, rock crab) experienced an apparent buffer from kelp decline following the demise of *Pycnopodia* (Eisaguirre et al., 2020). Finally, on the central coast of California, remnant patches of kelp forests were indirectly maintained by sea otters that target energetically profitable sea urchins in patches of forest (Smith et al., 2021). This spatially explicit foraging by sea otters is likely the mechanism responsible for the persistence of kelp patches within the mosaic.

In this study, switching among patch states within the mosaic was explicable in part by changes in the density of exposed (i.e. active foraging) sea urchins. Behavioural switching within the mosaic across such a short temporal duration may be driven by spatial variability in drift kelp. High levels of drift kelp have been shown to facilitate reef‐scale behavioural feedback in California, Chile and New Zealand (Karatayev et al., 2021; Kriegisch et al., 2019; Ling et al., 2019; Vásquez & Buschmann, 1997). We also found evidence of strong discontinuous state shift thresholds, with at least two discontinuous thresholds required to facilitate switching among patch states. A number of studies have suggested a critical threshold of a forcing variable that drives state transitions to less productive configurations (Casini et al., 2009; Petraitis & Dudgeon, 2004). The strong forward‐ and reverse‐shift thresholds identified in this study provide an empirical demonstration of this phenomenon.

Sea urchin movement from deep to shallow water may explain the isolated recovery of kelp forest patches in deep water. The dramatic reduction in medium‐ and large‐sized urchins at deep reefs, simultaneous increase of those size classes inshore and the pronounced reduction of foliose red macroalgae in shallow water all indicate that sea urchin movement is one possible explanation for the observed changes in the cover of macroalgae. Although other studies have documented sea urchin migrations between depth zones (Ling et al., 2016; Vadas et al., 1986), an alternative explanation in this system is that sea urchins occupying the deep reefs switched to a passive‐grazing modality and those in the shallow zone emerged from the refuge. However, because there was not a reduction of macroalgae in the shallow zone prior to the increase in the density of medium‐ and large‐sized urchins, the movement hypothesis (as opposed to behavioural switching) remains the most parsimonious explanation for observed recovery dynamics.

At the locations where kelp recovery was observed, it is important to note the kelp species that repatriated the once barren grounds was the bull kelp (*Nereocystis luetkeana*, a predominately annual species), not the giant kelp (*Macrocystis pyrifera*, a perennial species). Prior to the 2014 sea urchin outbreak, kelp forests along the Monterey Peninsula were dominated by the giant kelp (Foster & Schiel, 1985; Graham et al., 1997). It is well established that shading by giant kelp limits algal recruitment and the growth of other non‐calcareous species (Kennelly, 1989; Reed & Foster, 1984). The removal of long‐standing giant kelp forests by purple sea urchin grazing may have released *Nereocystis* from light limitation, thereby enabling the rapid recolonisation and growth of *Nereocystis* following sea urchin movement inshore to shallow water.

The results presented in this study highlight the role of behaviorally mediated effects in structuring ecological communities. One of the most unusual aspects of this system is the ability of sea urchins to persist in low‐resource environments for extended periods of time (Ebert, 1967; Ebert, 1982; Smith & Garcia, 2021), which may contribute to the longevity of the alternative barrens state of the ecosystem. Therefore, the behaviour of grazers, especially ecosystem engineers, is fundamental to community and ecosystem dynamics.

## AUTHORS CONTRIBUTION

JGS conceived the study, performed the analyses and wrote the first draft of the manuscript. MTT developed the population state dynamics model, contributed to manuscript and model revisions and provided substantial conceptual input.

### PEER REVIEW

The peer review history for this article is available at https://publons.com/publon/10.1111/ele.14064.

## Supporting information


Appendix S1

## Data Availability

Data and source code are available on GitHub (https://github.com/joshgsmith/PatchDynamics).
