# Supplementary material for: Alternations in the foraging behaviour of a primary consumer drive patch transition dynamics in a temperate rocky reef ecosystem
Source: Ecol Lett. 2022 Jun 29;25(8):1827–38. doi: 10.1111/ele.14064 (PMC9546210; doi:10.1111/ele.14064)
Supplement: Supplementary file 1 — Appendix S1 [file ELE-25-1827-s001.pdf]

Supplementary Information Appendix

"Alternations in the foraging behavior of a primary consumer drives patch transition dynamics in a temperate rocky reef ecosystem"

Joshua G. Smith[1*,2], M. Tim Tinker[1]

1 – Department of Ecology and Evolutionary Biology, University of California, Santa Cruz, USA
2 – National Center for Ecological Analysis and Synthesis, University of California, Santa Barbara, USA

* Corresponding author: JoshSmith@nceas.ucsb.edu

Supplementary Information Appendix. Supplementary Methods

Population model overview

We used a stage-structured matrix model (Caswell 2001) to describe demographic transitions and population dynamics for purple sea urchins (*Strongylocentrotus purpuratus,* hereafter, 'sea urchins') during the study period (1999 – 2021) around the Monterey Peninsula, California, USA (Figure S1). We classified sea urchins based on test diameter, with 10 stages corresponding to 1 cm size increments: stage 1 = urchins ≤ 1 cm and stage 10 = urchins >9 cm. Recruitment of larval sea urchins into stage 1 was assumed to occur annually, and the annual per-capita probabilities of transition between all other stages was determined by annual size-specific rates of survival and growth, as described below. Sea urchins were also assumed to have size-specific probabilities of detection during scuba-based surveys. We developed a process model that used a hierarchical (multilevel) analytical approach to incorporate temporal variation in recruitment, survival, and detection probability, and we fit this model to two data sets using Bayesian Markov Chain Monte Carlo (MCMC) methods: 1) survey estimates of sea urchin density (mean number per transect), and 2) samples of relative abundance by size class.

Population state dynamics

To estimate size-specific growth transition rates, we used a Tanaka (1982) growth function within a simulation-based analysis adapted from Burt et al. 2018 to model the stochastic growth rates of *Strongylocentrotus purpuratus*. For a sea urchin at year t with a specified diameter D_t, we calculate the expected diameter of the following year (D_{t+1}) as:

$$D_{t+1} = \frac{1}{\sqrt{f}}\log\left[\left(2f\left(\frac{E}{4f} - \frac{a}{E} + 1\right) + 2\sqrt{f^2\left(\frac{E}{4f} - \frac{a}{E} + 1\right) + f * a}\right)\right] + d$$

$$E = e^{\sqrt{f}(D_t - d)}$$

and the expected mean growth increment for a sea urchin of size D is calculated as the difference between D_{t+1} and D_t. We parameterized the Tanaka function growth function (parameters a, d, f) using data from the closest recorded location to our study area (Bodega Bay, California, USA) as reported by Ebert (2010). Individual variance in growth increment (σ_G^2) was not reported directly by Ebert (2010), however in Figure 7 of that paper the 95% confidence interval is provided for the mean expected growth increment for a 3 cm sea urchin. Based on the reported mean growth increment for Bodega Bay sample (0.525 cm), the 95% CI of the mean (+/- 0.05 cm) and the sample size (n = 141), we back calculated the value of σ_G^2 as 0.09 cm. We used the mean and variance in expected growth increments to parameterize size-specific gamma distributions describing individual variation in growth increments. We then iteratively calculated stochastic growth increments for the empirical size distribution of sea urchins in Monterey and used these to compute growth transition probabilities. Specifically, we conducted 3 steps for each of 10,000 simulations: 1) we initialized sizes at time t by distributing recorded discreet size classes randomly across a 1 cm interval (e.g., size class 1 individuals were distributed randomly between 0.5001 cm and 1.4999 cm); 2) we sampled random growth increments for each sea urchin from gamma distributions with variance σ_G^2 and mean values calculated using the Tanaka function growth function (Figure S2); 3) we computed the proportion of sea urchins in size class i at year t whose stochastic growth increment resulted in classification as size class i+1 at year t+1. We

then averaged these proportions across simulations to calculate size-specific transition probabilities (G_i) for the 10 size-classes of sea urchins (Figure S3), which we used to construct a growth transition matrix (Table T1).

The remaining demographic processes – recruitment and survival – as well as the probability of detection by scuba survey, were treated as parameters to be estimated by Bayesian methods. However, while we were interested in how the relative values of each of these parameters varied over time, we were unable to simultaneously estimate the absolute values of all three processes due to model identifiability limitations. We therefore fixed the value for minimum asymptotic detection probability to an arbitrary (and low) value and allowed all other parameters to vary. Annual recruitment (R_t) was assumed to be a stochastic process de-coupled from local abundance, whereby a number of new individuals were added to the first size class each year:

$$R_t = \exp\left(R_0 + \varepsilon_{R,t}\right)$$

where R_0 is the estimated mean value of log-transformed recruitment and $\varepsilon_{R,t}$ is a random effect that allows for year-to-year variation around the long-term mean value R_0, and is assumed to follow a normal distribution with mean of 0 and standard error σ_R. Based on the segmented regression analysis, we expected a breakpoint in population dynamics after 2013. However, recognizing that changes in recruitment during the years immediately prior to the temporal breakpoint may have contributed the observed increase after 2013, we adjusted the model to accommodate a change in variation of recruitment dynamics up to 2 years prior to the 2013 breakpoint. Specifically, we allowed for a shift in the magnitude of variance by fitting two variance parameters, σ_{R1} describing annual variance in recruitment from 1999-2011, and σ_{R2} describing annual variance in recruitment from 2012-2020.

Annual survival rates ($S_{i,t}$) were estimated as exp($-M_t$), where M_t is the instantaneous mortality rate at year t. We allowed for stochastic annual variation in annual mortality (and thus survival):

$$M_t = \exp\left(\gamma_0 - \varepsilon_{S,t}\right)$$

where γ_0 is the estimated mean log mortality rate, and $\varepsilon_{S,t}$ is a random effect allowing for variation across years, assumed to follow a normal distribution with mean of 0 and standard error terms σ_{S1} (for 1999-2013) or σ_{S2} (for 2014-2020). Based on previously published estimates of sea urchin survival (Ebert 2010), we assume a constant survival rate for all size classes >1 cm. Combining these demographic processes, we estimated the expected number of individuals in each size class at year t ($N_{i,t}$) using a series of recursive growth equations:

$$N_{1,t} = [N_{1,t-1} \cdot G_{1,1} + R_t] \cdot S_{1,t}$$
$$N_{2,t} = [N_{2,t-1} \cdot G_{2,2} + N_{1,t-1} \cdot G_{2,1}] \cdot S_{2,t}$$
$$\cdots$$
$$N_{10,t} = [N_{10,t-1} + N_{9,t-1} \cdot G_{10,9}] \cdot S_{10,t}$$

We incorporated these equations into stage-based projection matrix **A** (Table T2) to facilitate computation of annual population dynamics. For each possible set of parameter values, we initialized the population vector at year 0 (<$N_{1,0}$, $N_{2,0}$, … $N_{10,0}$>, where year 0 = 1998) via iterative projection, repeatedly taking the matrix product of matrix **A** and the population vector until the projected abundance of each size class stabilized at equilibrium values. We thus assumed that the system was approximately at equilibrium (i.e., stochastically fluctuating around average baseline values) at the start of the study period. The abundance of each size class in subsequent years ($N_{i,t}$, where t = 1, 2, …T) was then calculated by recursive matrix projection, such that the abundance at year t depended on the abundance at t-1 as well as the annual stochastic rates of recruitment and survival.

The mean number of urchins of each size class observed on transects each year ($Obs_{i,t}$) was assumed to depend both on true abundance ($N_{i,t}$) and on the probability that urchins of each size class at year t were detected ($P_{i,t}$), given their behavior. Previous analyses (Burt et al. 2018) have shown that detection probability is very low for the smallest size classes and increases asymptotically for larger size classes. Accordingly, we modeled the average size-specific detection probability as a non-linear asymptotic logit function:

$$logit(P_{i,t}) = \alpha - \beta\left(\frac{1}{i}\right)^2 + \varepsilon_{P,t}$$

where parameters α and β determine the shape and scale of the function and the $\varepsilon_{P,t}$ term is a random effect allowing for temporal variation in detection probability, assumed to follow a normal distribution with mean of 0. To ensure model identifiability we fixed α at -3.91, thus forcing an initial asymptotic baseline value for minimum detection probability of 0.02. The magnitude of variation in logit detection probability was determined by standard error parameters σ_{P1} (for 1999-2013) or σ_{P2} (for 2014-2020). The total expected density of sea urchins in year t (units of sea urchins per transect) was calculated as:

$$Density.Exp_t = \sum_i N_{i,t} \cdot P_{i,t}$$

and the expected proportions of detectable sea urchins in each size class at year t (a vector with length 10) were calculated as:

$$Distrib.Exp_t = {N_{i,t} \cdot P_{i,t}} \Big/ {\sum_i N_{i,t} \cdot P_{i,t}}$$

Model Fitting Methods

To fit the model, we compared the expected values of total detectable sea urchins per transect and the relative abundance of detectable sea urchins in each size class (i.e., the estimates associated with a particular set of parameter values) with the observed survey counts and size class distributions. In the case of mean sea urchin density, we assumed that observer error (i.e., variation in counts attributable to sampling error and measurement uncertainty) was described by a gamma distribution:

$$Density.Obs_t \sim gamma(Density.Exp_t \cdot \varphi, \varphi)$$

where φ is an inverse scale parameter that determines the magnitude of observer error, and the mean value of the gamma distribution corresponds to the expected value projected by the model. We note that observed sea urchin density data were available for the entire study period (1999 – 2021), while observed data on size class distributions were only available for the years 2011 – 2021. In the case of size class data, we assumed the proportion of measured urchins occurring in each size class in year t (a vector with length 10) could be described by a Dirichlet distribution:

$$Distrib.Obs_t \sim Dirichlet\left(Distrib.Exp_t \cdot [\, \theta_1 \cdot n_t{}^{\theta_2}]\right)$$

where the term $[\, \theta_1 \cdot n_t{}^{\theta_2}]$ determines the relative precision of the expected frequency distributions, calculated as an increasing but asymptotic power function of the number of urchins measured in year t (n_t), with intercept and slope determined by fitted parameters θ_1 and θ_2.

The observed data sets constrain the possible values of unknown parameters in the process model (σ_{R1}, σ_{R2}, σ_{S1}, σ_{S2}, σ_{P1}, σ_{P2}, R_0, γ_0, β, φ, θ_1 and θ_2), as well as hierarchical random effects ($\varepsilon_{R,t}$, $\varepsilon_{S,t}$, $\varepsilon_{P,t}$), allowing us to estimate posterior distributions for these parameters using standard Markov Chain Monte Carlo (MCMC) methods. We used R (R.Core.Team 2014) and Stan software (Carpenter et al. 2017) to code and fit the model, saving 10,000 samples after a burn-in of 2,000 samples. We used Cauchy(0, 2.5) prior distributions for unconstrained parameters R_0 and γ_0, and for all remaining parameters (which were biologically or mathematically constrained to be positive) we used half-Cauchy(0, 2.5) prior distributions, as these distributions have been shown to ensure appropriate shrinkage for non-significant parameters while allowing for unbiased estimates for significant parameters (Gelman et al. 2008). We evaluated model convergence by graphical examination of trace plots from 10 independent chains and by ensuring that Gelman-Rubin convergence diagnostic (r-hat) was ≤1.05 for all fitted model parameters. We conducted posterior predictive checking (PPC) to evaluate model goodness of fit, both by graphical comparison of the distributions of empirical data vs. out-of-sample ("new") estimates, and by using the χ^2 statistic (sum of squared Pearson residuals for observed counts vs expected values) to compare fit of observed data and out-of-sample estimates (Gelman et al. 2000). We examined scatter plots of the posterior distribution of χ^2 scores for new vs observed data (in the case of well-fitting models, points in such a plot should be distributed around a line with slope 1) and we computed the associated "Bayesian-P" value (the proportion of new observations more extreme than existing observations; Gelman 2005, Ghosh et al. 2007), which should fall within the range 0.2< Bayesian-P <0.8 for a well-fit model. We evaluated model identifiability by graphically comparing posterior vs. prior density distributions for all parameters. For random effects that described temporal variation in survival ($\varepsilon_{S,t}$), recruitment ($\varepsilon_{R,t}$), and detection probability ($\varepsilon_{P,t}$), we calculated shrinkage factors (*SF*) as a means of further evaluating parameter identifiability. The *SF* statistic measures the degree to which individual levels of a hierarchical random effect are distinct from the population mean, and ranges from a value of 1 (no influence of the population mean on individual effect levels) to 0 (individual effect levels are pooled together, converging on the population mean). We calculated *SF* statistics from posterior distributions using standard methods (Gelman and Pardoe 2006), and we conducted separate analyses for before vs. after the temporal break. Finally, to assess the degree to which temporal variation in each process was supported by the available data, we computed and plotted

likelihood profiles for each of the variance parameters (σ_R, σ_S, σ_P). We did this by sequentially holding one of the variance parameters fixed (at values ranging from 0 to 100% of the true estimates), re-fitting the model, and computing the associated log likelihood. We calculated log likelihoods as the expected log predictive density (*elpd*) estimated from full posteriors using the "Loo" package in R (Vehtari et al. 2017).

We summarized results graphically and by reporting the mean and 90% credible intervals (CI) of posterior distributions for base parameters. We plotted the observed vs. hindcast trends in urchin counts and size distributions over the study period. We also calculated and report on the posterior distributions for 3 derived parameters, Δ_R, Δ_S, Δ_P, which were calculated as the difference in mean log-transformed estimates of annual recruitment, survival, and detection probability (respectively) before vs. after the 2014 temporal breakpoint. For survival, and detection probability we compared estimates for 2015-2020 vs. 2000-2013 (i.e., excluding the 2014 breakpoint year and the first and last years of the time series), while for recruitment we compared 2012-2020 vs. 2000-2011 (recognizing that increased recruitment 1-2 years prior to the temporal breakpoint may have contributed to the initial increase in measured urchins). These Δ parameters can be interpreted as the log of the mean proportional change in each variable, with positive values indicating an increase after the temporal breakpoint. In the case of Δ_P we calculated the proportional change in detection of the largest size class of sea urchins, noting that the proportional change for smaller size classes would be even greater.

Finally, to evaluate the relative contribution of changes in recruitment, survival, and detection probability to the observed increase in sea urchin counts after 2013, we conducted a simulation-based sensitivity analysis. Specifically, we iterated hindcast simulations of population dynamics and expected urchin counts, drawing randomly from the joint posterior distributions of all parameters and random effects, and for each simulation we calculated Δ_C as the proportional change in mean expected density in 2015-2020 vs. mean expected density in 2000-2013. We then repeated this procedure allowing for observed random effects in just one of the key processes (R_t, S_t, $P_{i,t}$), while the other two processes were constrained to pre-2014 levels (by drawing randomly from distributions for 2000-2010), and again calculated the proportional change in mean expected density in 2015-2020 vs. the mean expected density in 2000-2013 ($\Delta_{C,R}$, $\Delta_{C,S}$, $\Delta_{C,P}$, for simulations allowing for observed variation in R_t, S_t, or $P_{i,t}$, respectively). We then calculated the relative impacts of increased variation in recruitment, survival, and detection probability (respectively) as $\Delta_{C,R}/\Delta_C$, $\Delta_{C,S}/\Delta_C$, and $\Delta_{C,P}/\Delta_C$.

Model Fitting Results

The model converged well (r-hat values for all parameters <1.05; Table S3) and provided excellent goodness of fit (Figure S4). The Bayesian-P value was 0.493, indicating that out-of-sample model predictions were in good agreement with observed data. For most model parameters there was a clear distinction between posterior distributions and the Cauchy prior distributions (Figure S5) consistent with parameter identifiability given the available data. The shrinkage factor (*SF*) estimates indicate that random effect levels were distinct from the long-term mean value for detection probability, less distinct for recruitment, and were pooled close to the mean value for survival (Table S3). In all cases there was an increase in *SF* values after the temporal breakpoint, suggesting increased support for year-to-year differences after 2014. Likelihood profiles calculated for the three variance parameters indicated a substantial reduction

in model likelihood as variance in detection probability (σ_P) was reduced towards 0, but only minimal reductions in likelihood associated with reduced values of σ_R and σ_S (Figure S6). Model-estimated dynamics were consistent with both the observed trends in urchin abundance (main manuscript, Figure 2a) and the year-to-year differences in size distribution (Figure S7). Our results indicate significant increases in both recruitment and detection probability over the study period (main manuscript, Figure 2b and 2c): mean detection probability for large urchins increased 37-fold after 2013 (Δ_P = 3.620, CI_{90} = 3.343 – 3.858), while there was a 5-fold increase in recruitment (Δ_R = 1.708, CI_{90} = 1.174 – 2.243) and no significant directional change in survival (Figure S8). The results of a simulation-based sensitivity analysis indicate that increased detection probability explained approximately 93% of the observed increase in urchin abundance after 2014, while variation in recruitment explained 5.6% and survival variation just 1.2% of the increase (main manuscript, Figure 2d).

Effects of reef topography on counts of sea urchins

We tested for a relationship between sea urchin counts and terrain ruggedness (i.e., reef complexity), reef slope, and relief to determine whether these variables influenced observed increases in sea urchin abundance at each PISCO site. We used seafloor mapping data available on a public server at California State University, Monterey Bay to derive estimates of terrain ruggedness and relief slope. We regressed the total counts of sea urchins from each site against the average terrain ruggedness, reef slope, and diver estimates of relief (Malone et al. 2021). Results indicated that these substrate variables were not significant predictors of variation in counts in sea urchins. Moreover, refuge availability was relatively uniform across sampled sites.

Supplementary Information Appendix. Supplementary Tables

Table T1. Growth transition probabilities for 10 size-classes of sea urchins calculated using a Tanaka growth function and model parameters derived from Ebert 2010.

G =

	i_1	i_2	i_3	i_4	i_5	i_6	i_7	i_8	i_9
i_1	0.1046	0	0	0	0	0	0	0	0
i_2	0.8954	0.0705	0	0	0	0	0	0	0
i_3	0	0.9294	0.4040	0	0	0	0	0	0
i_4	0	0	0.5959	0.7499	0	0	0	0	0
i_5	0	0	0	0.2500	0.9147	0	0	0	0
i_6	0	0	0	0	0.0852	0.9147	0	0	0
i_7	0	0	0	0	0	0.0852	0.9149	0	0
i_8	0	0	0	0	0	0	0.0850	0.9145	0
i_9	0	0	0	0	0	0	0	0.0854	0.9167
i_{10}	0	0	0	0	0	0	0	0	0.0832

Table T2. Stage-based projection matrix used to calculate annual demographic transitions for a purple urchin population in which individuals are classified into 10 size classes.

A =

	i_1	i_2	i_3	i_4	i_5	i_6	i_7	i_8	i_9	i_{10}
i_1	$\left(G_{1,1} + \frac{R_t}{N_{1,t-1}}\right) \cdot S_{1,t}$	0	0	0	0	0	0	0	0	0
i_2	$G_{2,1} \cdot S_{2,t}$	$G_{2,2} \cdot S_{2,t}$	0	0	0	0	0	0	0	0
i_3	0	$G_{3,2} \cdot S_{3,t}$	$G_{3,3} \cdot S_{3,t}$	0	0	0	0	0	0	0
i_4	0	0	$G_{4,3} \cdot S_{4,t}$	$G_{4,4} \cdot S_{4,t}$	0	0	0	0	0	0
i_5	0	0	0	$G_{5,4} \cdot S_{5,t}$	$G_{5,5} \cdot S_{5,t}$	0	0	0	0	0
i_6	0	0	0	0	$G_{6,5} \cdot S_{6,t}$	$G_{6,6} \cdot S_{6,t}$	0	0	0	0
i_7	0	0	0	0	0	$G_{7,6} \cdot S_{7,t}$	$G_{7,7} \cdot S_{7,t}$	0	0	0
i_8	0	0	0	0	0	0	$G_{8,7} \cdot S_{8,t}$	$G_{8,8} \cdot S_{8,t}$	0	0
i_9	0	0	0	0	0	0	0	$G_{9,8} \cdot S_{9,t}$	$G_{9,9} \cdot S_{9,t}$	0
i_{10}	0	0	0	0	0	0	0	0	$G_{10,9} \cdot S_{10,t}$	$S_{10,t}$

Table T3. Summary of parameter estimates from the best-supported model of sea urchin population dynamics. The posterior distributions of each parameter are described by the mean, standard deviation (sd), 5% and 95% quantiles, the effective sample size (N_eff), and the r-hat statistic (which measures chain convergence: values should be <1.05). In the case of random effect parameters ($\varepsilon_{R,t}$, $\varepsilon_{S,t}$, $\varepsilon_{P,t}$) we summarize values for years pre- and post-temporal break, reporting average values of sd, N_eff and r-hat for each period and also reporting the shrinkage factor (*SF*) associated with effect level estimates within each period.

Parameter	Name	Description	*SF*	mean	sd	q5	q95	N_eff	rhat
Bayesian-P	ppp	Posterior predictive check		0.493	0.500	0.000	1.000	7982.5	1.000
σ_{R1}	sigR[1]	Variance recruitment, period 1		3.460	1.632	0.643	6.203	526.2	1.017
σ_{R2}	sigR[2]	Variance recruitment, period 2		0.716	0.605	0.158	1.998	486.5	1.018
σ_{S1}	sigS[1]	Variance survival, period 1		1.544	0.938	0.245	3.208	2190.7	1.003
σ_{S2}	sigS[2]	Variance survival, period 2		2.179	1.224	0.775	4.443	2354.5	1.003
σ_{P1}	sigP[1]	Variance detection probability, period 1		0.443	0.229	0.062	0.822	1188.2	1.008
σ_{P2}	sigP[2]	Variance detection probability, period 2		6.822	2.372	3.944	11.389	1163.3	1.007
R_0	R0	Baseline log recruitment rate		5.186	0.614	3.806	5.825	544.9	1.017
γ_0	gamma0	Baseline log hazard rate		-3.002	0.589	-4.158	-2.209	3646.5	1.002
β	Beta	Size effect on logit detection probability		10.871	1.355	8.558	13.052	2629.1	1.004
φ	phi	inverse scale parameter, gamma-distributed desnities		3.902	3.870	0.706	11.984	956.7	1.016
θ_1	theta[1]	parameter 1, sample size effect on precision of Dirichlet-distributed size frequencies		5.002	2.659	1.919	10.194	3790.7	1.002
θ_2	theta[2]	parameter 2, sample size effect on precision of Dirichlet-distributed size frequencies		0.284	0.062	0.180	0.384	3216.3	1.003
Δ_R	logRdff	log proportional differnce in mean recruitment rate, period 2 vs. period 1		1.708	0.329	1.174	2.243	4175.8	1.002
Δ_S	logSdff	log proportional differnce in mean survival rate, period 2 vs. period 1		0.001	0.061	-0.100	0.097	1378.7	1.009
Δ_P	logPdff	log proportional differnce in mean detection probability, period 2 vs. period 1		3.620	0.159	3.343	3.858	3877.6	1.004
ε_{R_pre}	epsR[1:13]	Random effects, recruitment, period 1	0.114		0.670			2869.8	1.003
ε_{R_post}	epsR[14:23]	Random effects, recruitment, period 2	0.391		0.725			2346.7	1.004
ε_{S_pre}	epsS[1:15]	Random effects, survival, period 1	0.026		0.973			7129.4	1.002
ε_{S_post}	epsS[16:23]	Random effects, survival, period 2	0.250		0.854			5711.5	1.001
ε_{P_pre}	epsP[1:15]	Random effects, detection, period 1	0.417		0.783			5153.9	1.002
ε_{P_post}	epsP[16:23]	Random effects, detection, period 2	0.698		0.280			1557.0	1.006

* Corresponding author: JoshSmith@nceas.ucsb.edu

Supplementary Information Appendix. Supplementary Figures

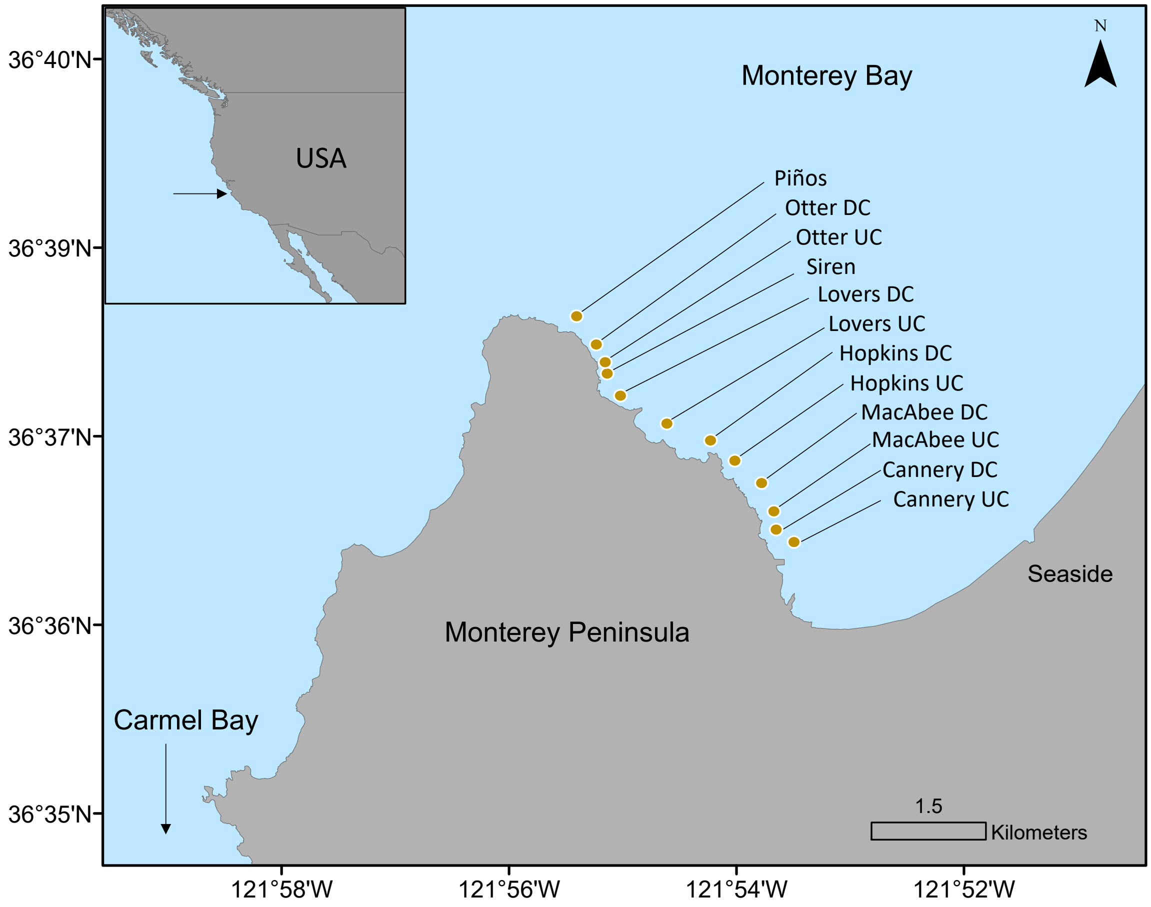

Figure S1. Approximate locations of the 12 PISCO long-term subtidal monitoring survey sites along the Monterey Peninsula, California, USA. Each site consisted of six replicate 2 m x 30 m transects stratified across three bottom depths (5 m, 12.5 m, 20 m; two transects per depth level). See Malone et al. 2021 for expanded description of sampling methods.

* Corresponding author: JoshSmith@nceas.ucsb.edu

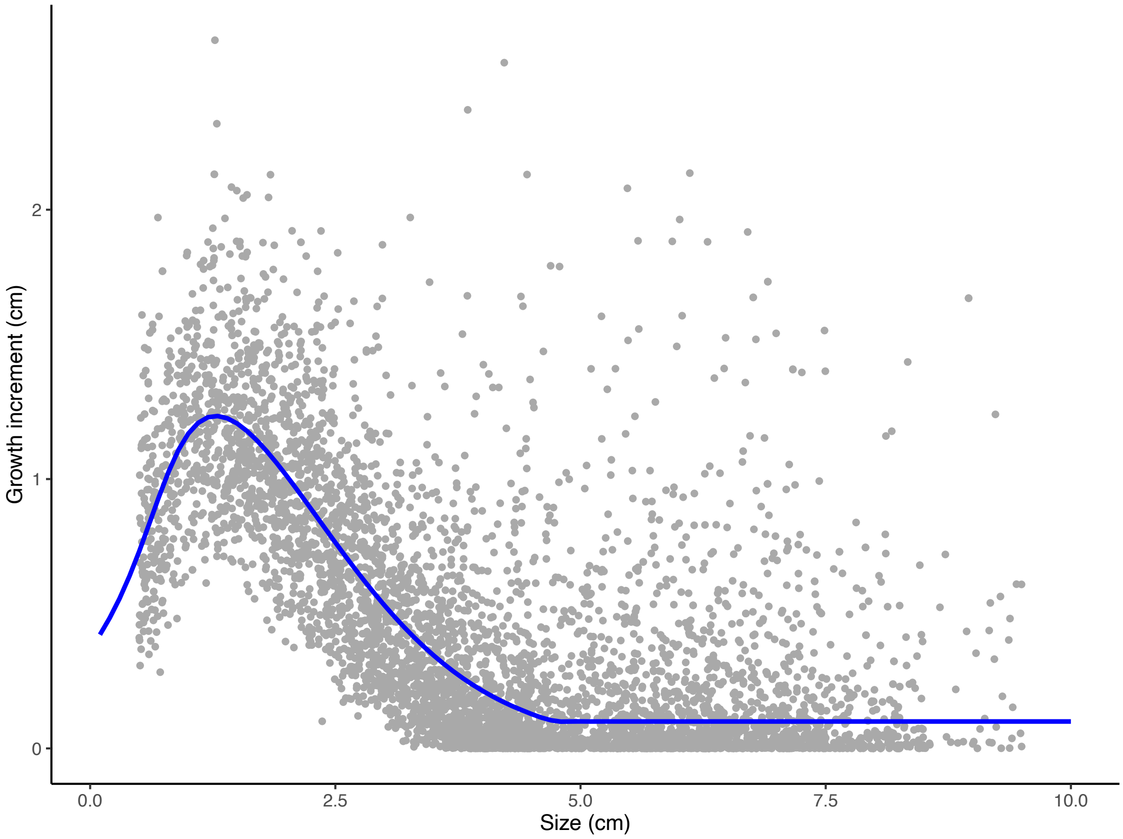

Figure S2. Simulated stochastic growth increments for purple sea urchins based on a random sample of starting sizes (from PISCO size sampling data, 2011-2020) and applying the Tanaka growth function with stochasticity. The blue line depicts a smoothed function of the expected growth increment t for each size class given deterministic application of the Tanaka growth function.

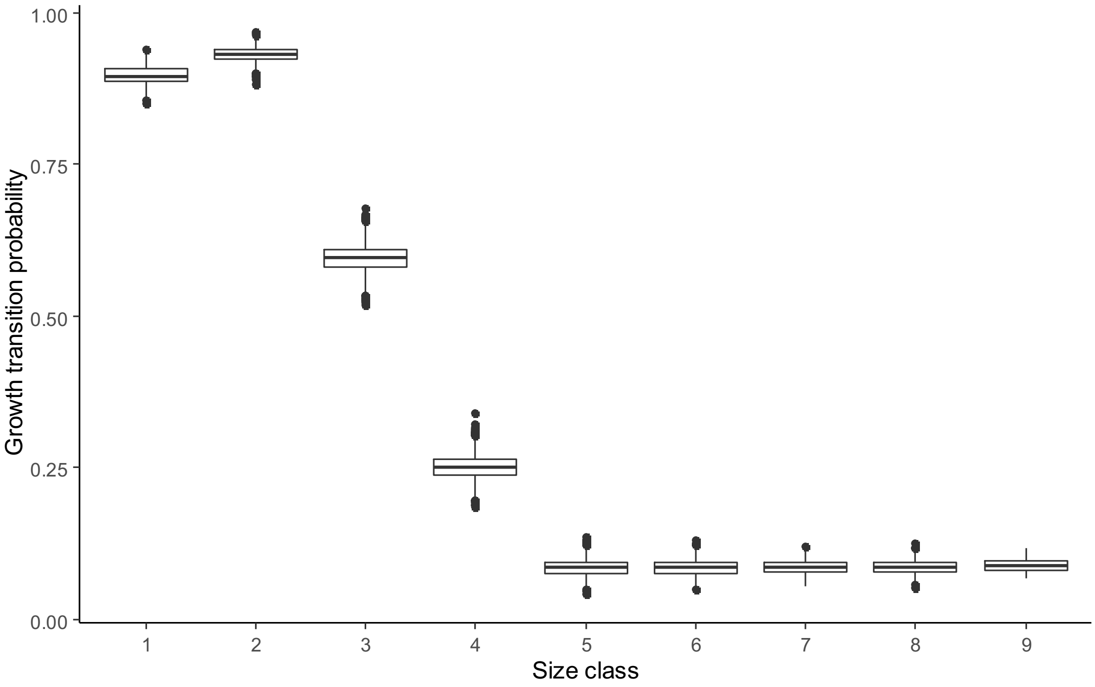

Figure S3. Estimated growth transition probabilities for 10 discrete sizes of sea urchins. Box plots depict the median value with interquartile range and whiskers indicate the distance to the furthest values.

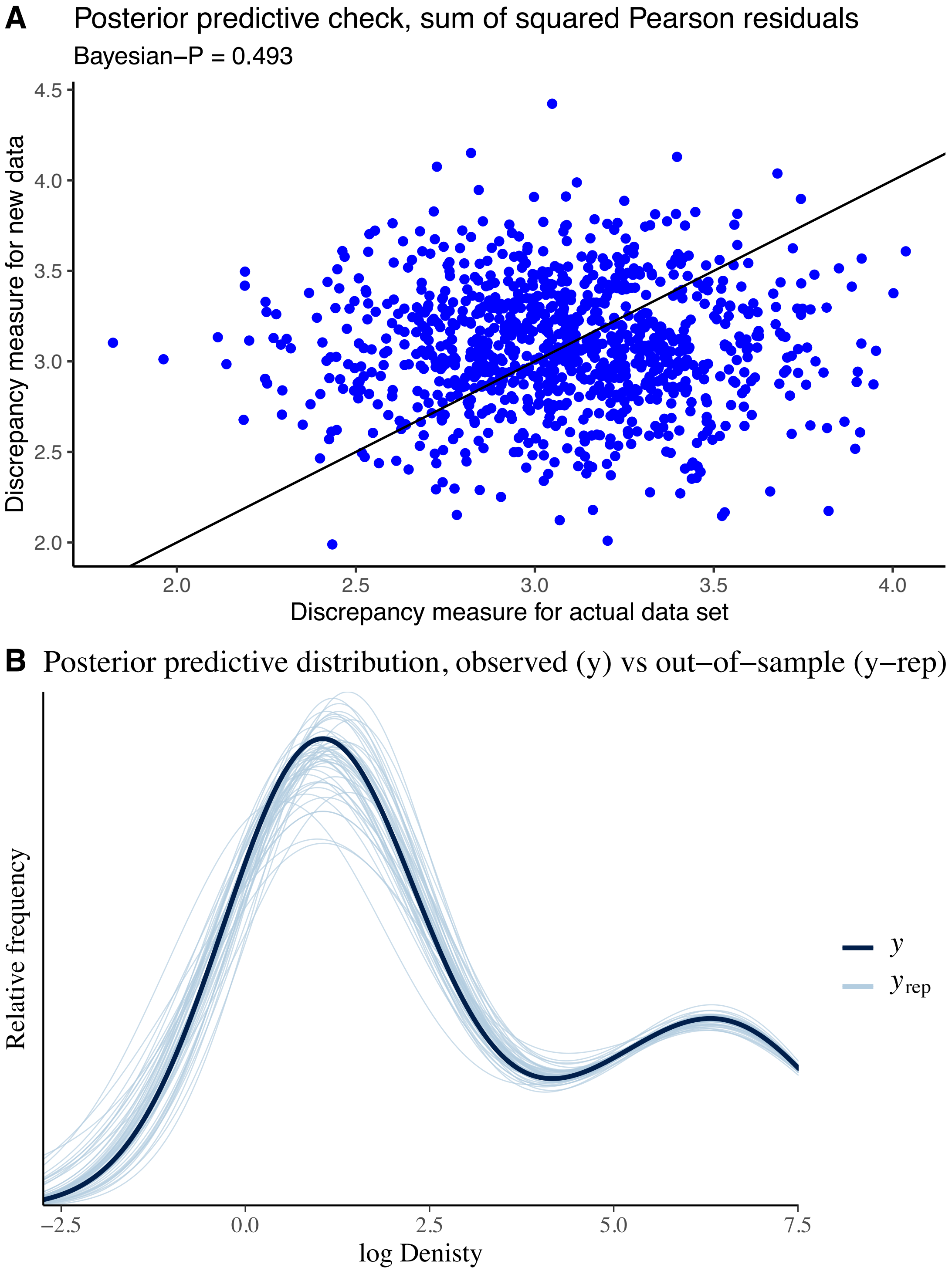

Figure S4. Graphical summaries of posterior predictive checks. A) scatter plot showing discrepancy measures (squared Pearson residuals) for "new", or out-of-sample model projections compared to observed data on survey counts of sea urchin abundance; B) frequency distribution of observed data ("y"; dark line) overlying a random selection of frequency distributions of out-of-sample predicted values ("y-rep"; light blue lines).

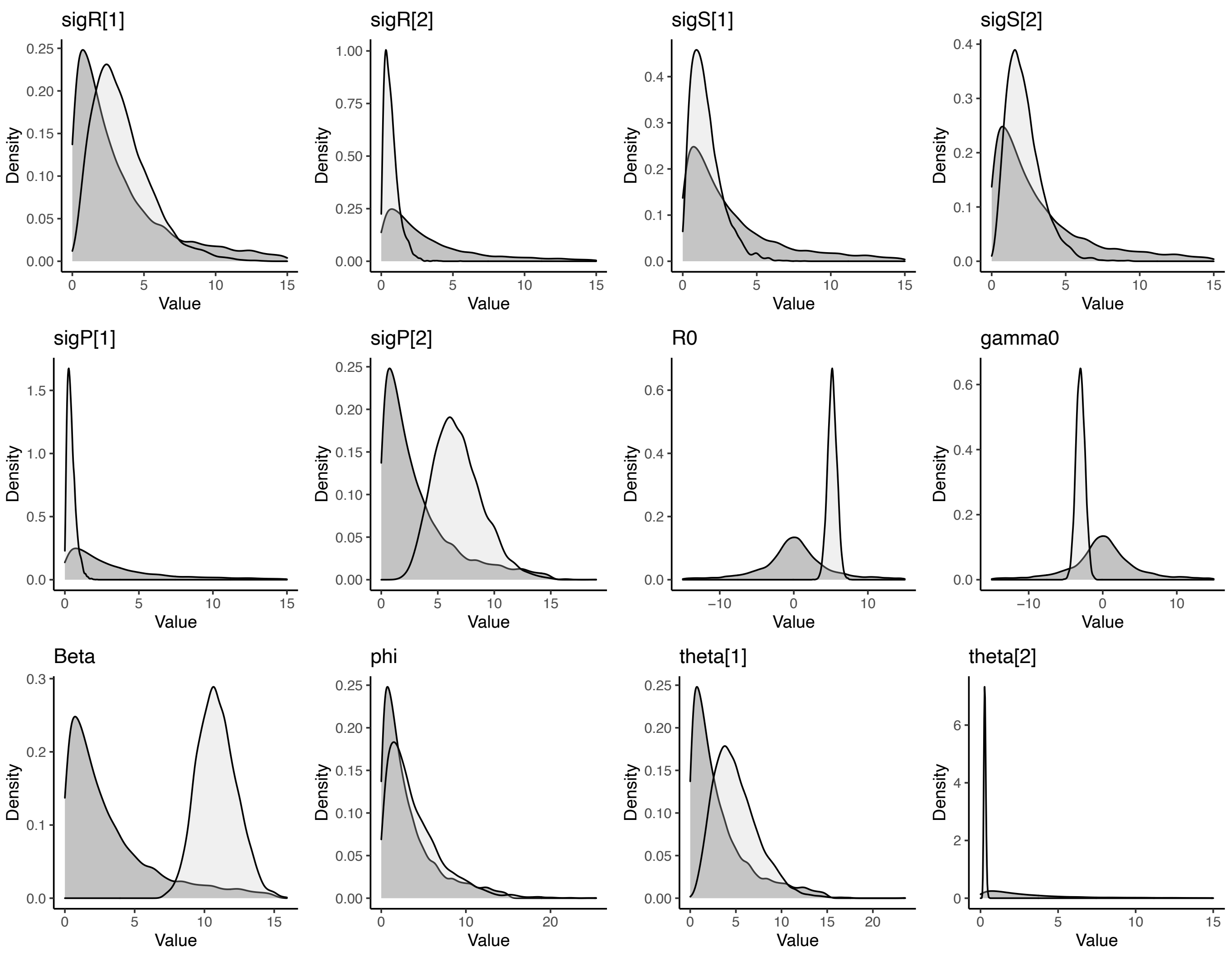

Figure S5. Density plots comparing the prior distribution (dark grey) and the fitted posterior distributions (light grey) for all base parameters in the model (refer to Table S3 for a description of each parameter). Note that all prior distributions are Cauchy or half-Cauchy distributions with parameters 0 and 2.5, which for plotting purposes have been constrained to the range of -15 to 15 (full Cauchy priors) or 0 to 15 (half-Cauchy priors)

* Corresponding author: JoshSmith@nceas.ucsb.edu

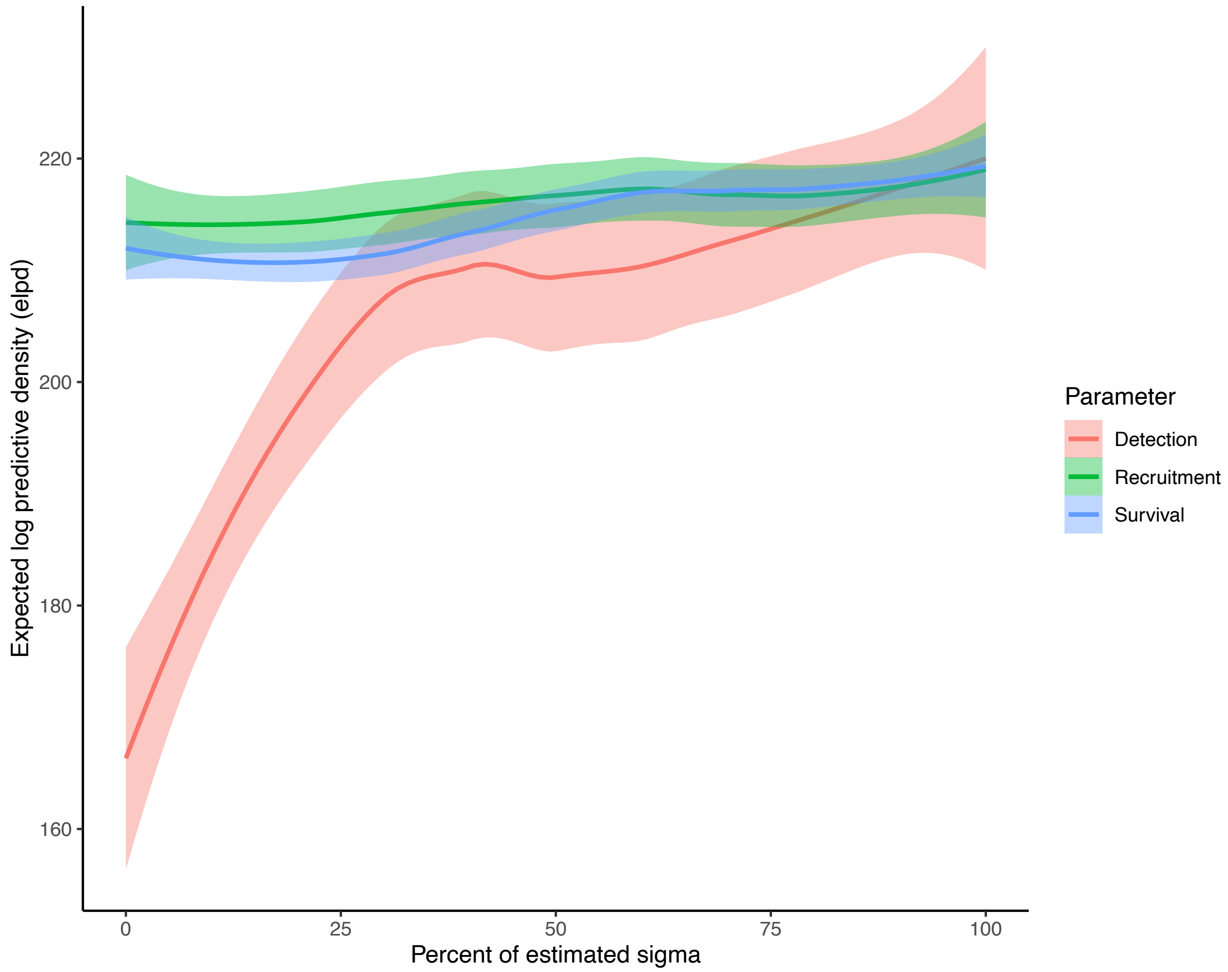

Figure S6. Likelihood profile plots illustrating how "forced" reductions in the temporal variation of recruitment (σ_R), survival (σ_S) and detection probability (σ_P) affect model likelihood, as measured by the expected log predictive density (*elpd*).

* Corresponding author: JoshSmith@nceas.ucsb.edu

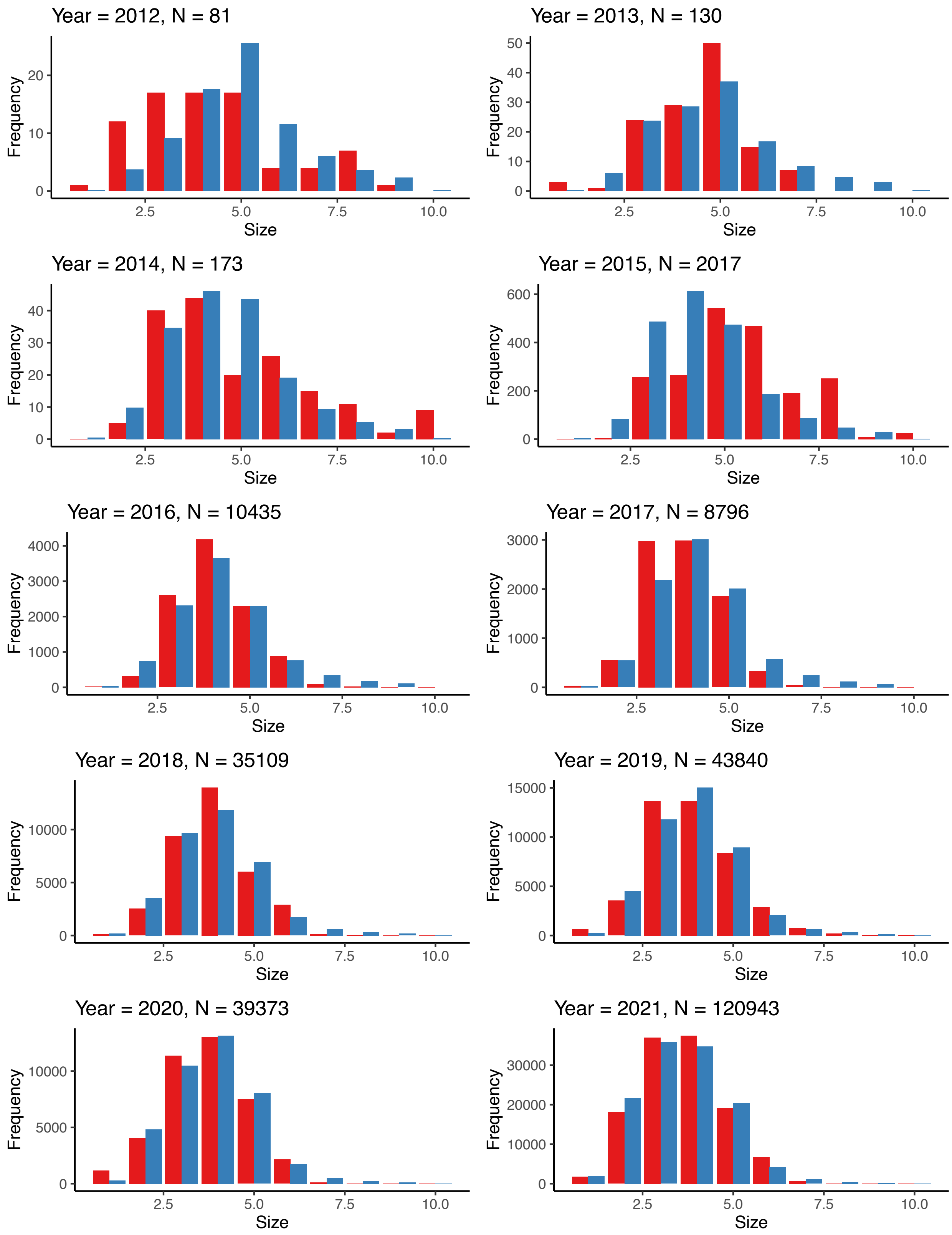

Figure S7. Histogram plots of the frequency of size-specific counts of urchins on transects between 2012 and 2021, comparing observed counts from scuba surveys (red) to model-predicted counts (blue).

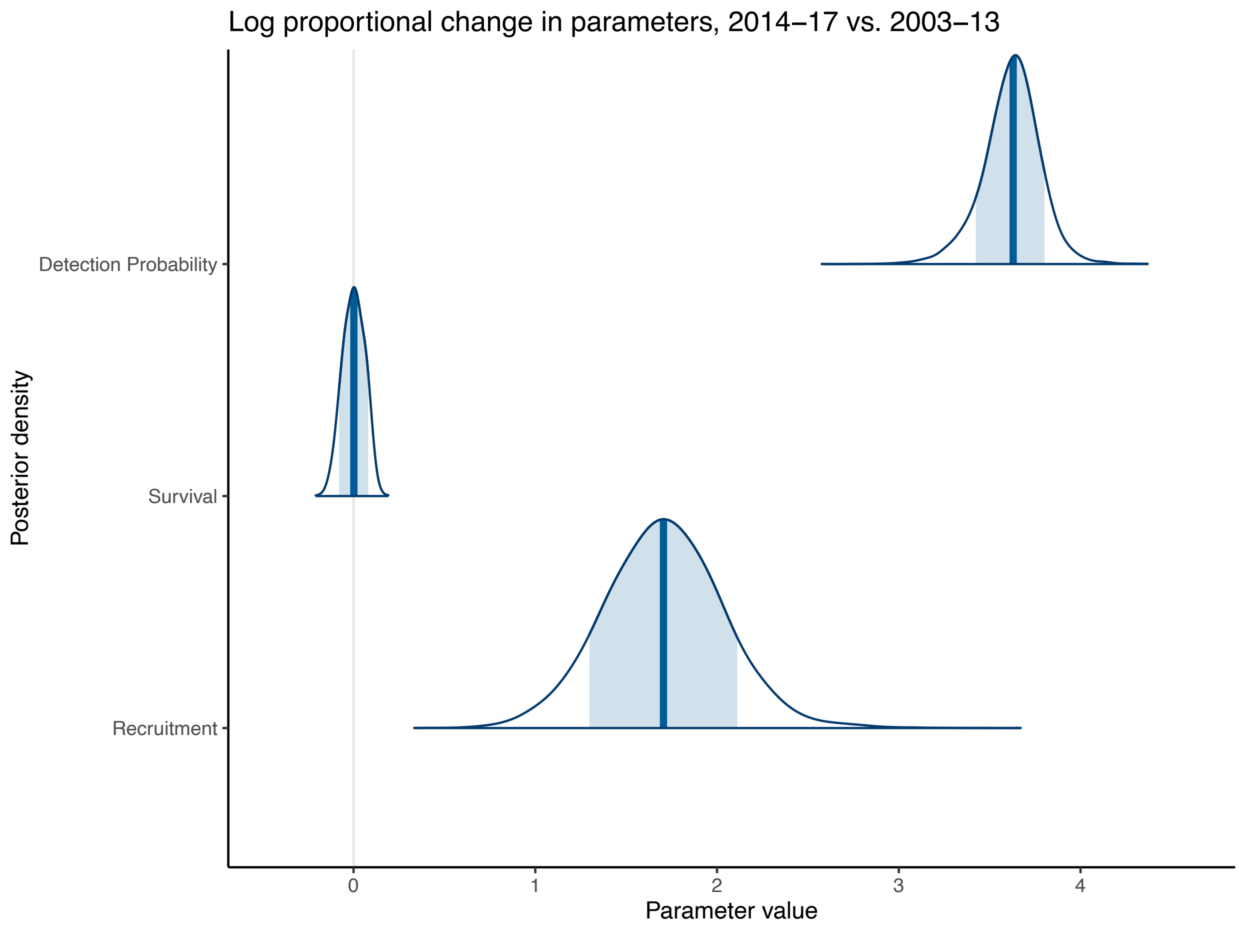

Figure S8. Posterior distributions for three derived parameters from the Bayesian sea urchin population model: Δ_R, Δ_S, Δ_P, which represent the difference in mean log-transformed estimates of annual recruitment, survival, and detection probability (respectively), after vs. before 2013.

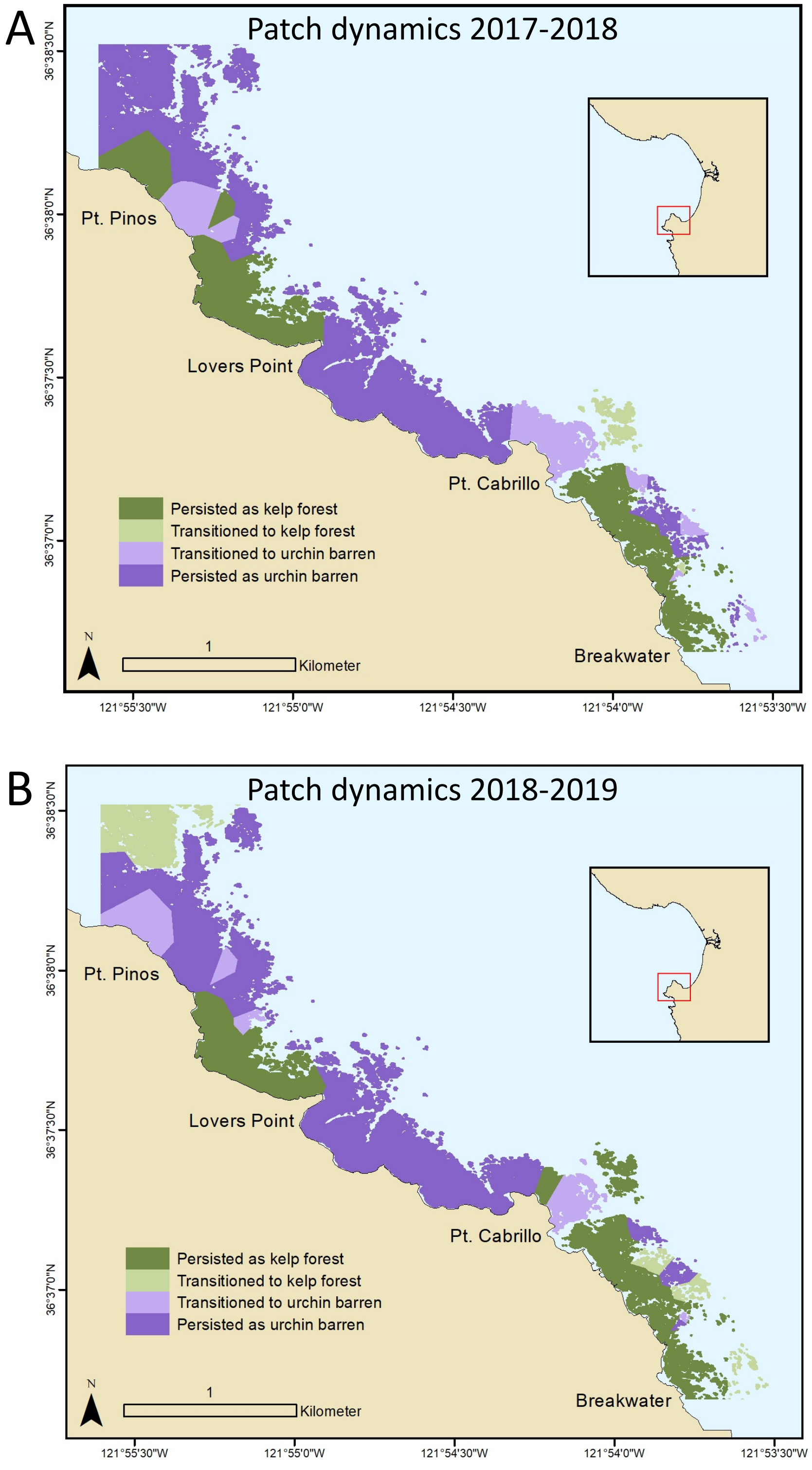

Figure S9. Patch transition dynamics across the 2017-2018 (A) and 2018-2019 (B) sampling periods interpolated using Thiessen polygons constrained to rocky reef substratum (Gullikson 2021).

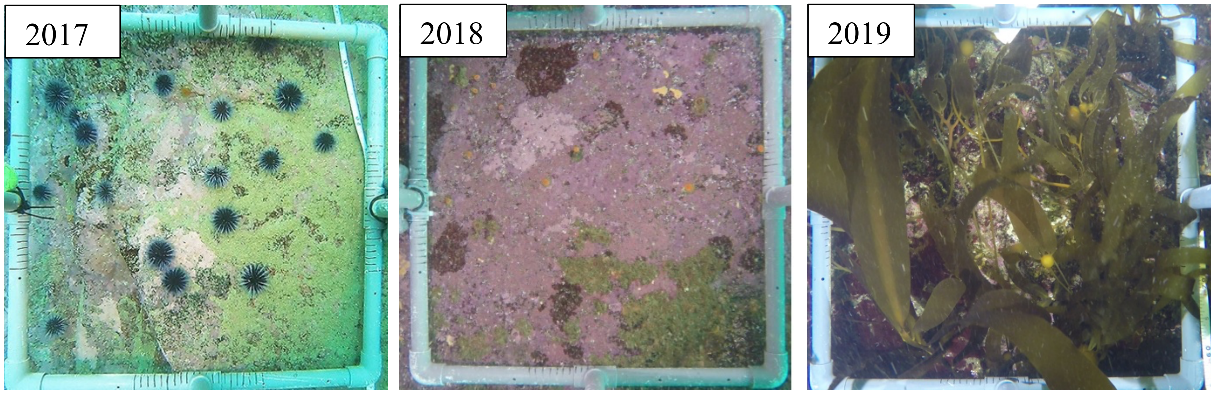

Figure S10. Representative photoquadrats from a deep water (20-meter) survey site sampled annually (2017-2019) at Pt. Pinos, CA, USA.

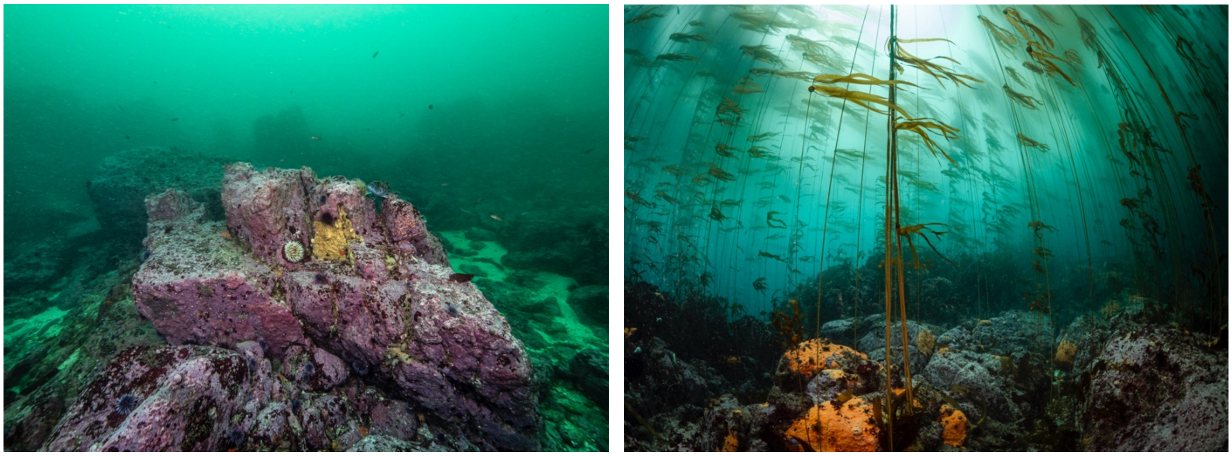

Figure S11. Landscape images of Pt. Pinos, CA, USA depicting the same reef devoid of macroalgae (left) and after recovery (right). Image credit: Patrick Webster.

References

Burt, J. M., Tinker, M. T., Okamoto, D. K., Demes, K. W., Holmes, K., & Salomon, A. K. (2018). Sudden collapse of a mesopredator reveals its complementary role in mediating rocky reef regime shifts. *Proceedings of the Royal Society B: Biological Sciences*, *285*(1883), 20180553.

Carpenter, B., A. Gelman, M. D. Hoffman, D. Lee, B. Goodrich, M. Betancourt, M. Brubaker, J. Guo, P. Li, and A. Riddell. 2017. Stan: A Probabilistic Programming Language. Journal of statistical software **76**:32.

Caswell, H. (2000). *Matrix population models* (Vol. 1). Sunderland, MA: Sinauer.

Ebert, T. A. 2010. Demographic patterns of the purple sea urchin Strongylocentrotus purpuratus along a latitudinal gradient, 1985–1987. Marine Ecology Progress Series **406**:105-120.

Gelman, A. 2005. Comment: Fuzzy and Bayesian p -Values and u -Values. Statist. Sci. **20**:380-381.

Gelman, A., Y. Goegebeur, F. Tuerlinckx, and I. Van Mechelen. 2000. Diagnostic checks for discrete data regression models using posterior predictive simulations. Journal of the Royal Statistical Society: Series C (Applied Statistics) **49**:247-268.

Gelman, A., A. Jakulin, M. G. Pittau, and Y.-S. Su. 2008. A weakly informative default prior distribution for logistic and other regression models. The Annals of Applied Statistics **2**:1360-1383.

Gelman, A., and I. Pardoe. 2006. Bayesian Measures of Explained Variance and Pooling in Multilevel (Hierarchical) Models. Technometrics **48**:241-251.

Ghosh, J. K., M. Delampady, and T. Samanta. 2007. An introduction to Bayesian analysis: theory and methods. Springer Science & Business Media, New York, NY.

Gullikson, L. 2021. Spatial extent of ecosystem patch dynamics following the outbreak of a benthic grazer. Undergraduate thesis submitted to the University of California, Santa Cruz.

Malone, D. P., Davis, K., Lonhart, S. I., Parsons-Field, A., Caselle, J. E., & Carr, M. H. (2021). Large scale, multi-decade monitoring data from kelp forest ecosystems in California and Oregon (USA). *Ecology*, e3630.

R.Core.Team. 2014. R: A language and environment for statistical computing. . R Foundation for Statistical Computing, Vienna, Austria.

Russell, M. P. (1987). Life history traits and resource allocation in the purple sea urchin Strongylocentrotus purpuratus (Stimpson). *Journal of Experimental Marine Biology and Ecology*, *108*(3), 199-216.

Tanaka, M. (1982). A new growth curve which expresses infinitive increase. *Publ. Amakusa Mar. Biol. Lab*, *6*(2), 167-177.

Vehtari, A., A. Gelman, and J. Gabry. 2017. Practical Bayesian model evaluation using leave-one-out cross-validation and WAIC. Statistics and Computing **27**:1413-1432.
